# Pharmacokinetics, absorption, distribution, metabolism and excretion of the MEK inhibitor zapnometinib in rats

**DOI:** 10.3389/fphar.2022.1050193

**Published:** 2022-12-05

**Authors:** Yvonne Füll, Christian Wallasch, Ashley Hilton, Oliver Planz

**Affiliations:** ^1^ Department of Immunology, Interfaculty Institute for Cell Biology, Eberhard Karls University of Tuebingen, Tuebingen, Germany; ^2^ Atriva Therapeutics GmbH, Tuebingen, Germany; ^3^ Labcorp Early Development Laboratories Ltd., Huntingdon, United Kingdom

**Keywords:** mek inhibitor, absorption, distribution, metabolism, excretion, antiviral therapy, zapnometinib

## Abstract

Zapnometinib is a MEK inhibitor currently under clinical development for the treatment of COVID-19 and influenza. Zapnometinib has both antiviral and immunomodulatory effects. Information concerning the absorption, distribution, metabolism, and excretion of the compound following single oral doses of 30 mg/kg [^14^C]-zapnometinib to rats was required to support pharmacology and toxicology studies in animals and clinical studies in man. As part of the development and safety assessment of this substance, zapnometinib was radioactively labeled and used for the investigation of time-dependent plasma concentrations, the rates and routes of excretion, the extent and time-course of compound distribution in body tissues, the metabolite profiles in plasma, urine and feces and the chemical nature of its metabolites. The present study reveals a rapid but low absorption of zapnometinib from the gastrointestinal tract, with more than 90% of the compound being excreted within 48 h, mainly *via* feces. Whole body autoradiography confirms that zapnometinib was rapidly and widely distributed, with greatest concentrations in the circulatory and visceral tissues. Maximum plasma and tissue concentrations occurred between two and 8 h post dose. Penetration into the brain was low, and elimination from most tissues almost complete after 168 h. Metabolic profiles showed that the main clearance routes were metabolism *via* oxidative reactions and glucuronidation. These results further strengthen the knowledge of zapnometinib with respect to the clinical development of the drug.

## Introduction

The mitogen-activated extracellular signal-related kinase (MEK) is a central component of the Raf/MEK/ERK signaling pathway, which belongs to the mitogen—activated protein kinase (MAPK) signaling cascades. The Raf/MEK/ERK signaling pathway is involved in the regulation of normal cell proliferation, survival, and differentiation. Dysregulation of the pathway has been shown to play an important role in the pathogenesis and progression of various cancers, making the components of this signaling cascade potentially important as therapeutic targets (Sebolt-Leopold 2004; Sebolt-Leopold and Herrera 2004; Friday and Adjei 2008).

CI-1040 (PD184352) (2-(2-chloro-4-iodo-phenylamino)-Ncyclopropylmethoxy-3, 4-difluoro-benzamide) was described as a potent and highly selective inhibitor of MEK with a 50% inhibitory concentration (IC_50_) of 17 nM (Lorusso et al., 2005). CI-1040 is an orally active, highly specific, small-molecule inhibitor and was developed until phase 2 clinical trial, but the development was terminated due to lack of antitumor response and low bioavailability (Lorusso et al., 2005). Analysis of the metabolites in cell lines and animal models demonstrated that CI-1040 undergoes extensive oxidative metabolism (14 metabolites identified). The hydroxylated metabolites were subsequently glucuronidated and excreted mainly through the bile *in vivo* (Wabnitz et al., 2004). A phase I and pharmacokinetic study of CI-1040 in patients with advanced malignancies demonstrated that plasma concentrations of an acid metabolite, named PD-0184264, were approximately 30-fold greater than those of CI-1040 (Lorusso et al., 2005). This favorable bioavailability led to the further development of PD-0184264, today known as zapnometinib.

**FIGURE 1:**
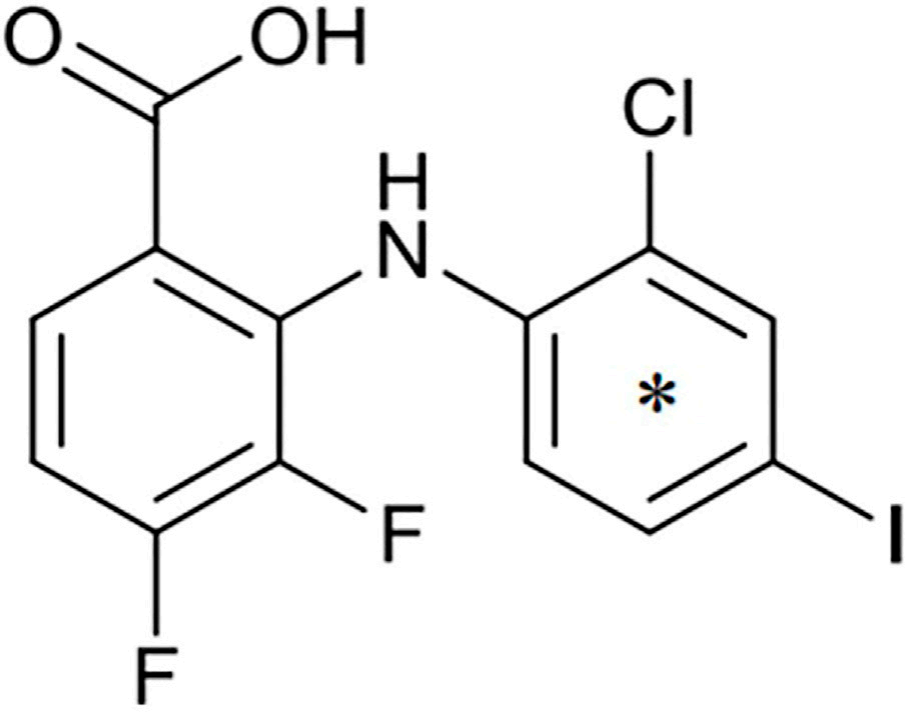
Structure of zapnometinib with position of ^14^C-radiolabel. Position of radiolabel is indicated by the asterisk (*).

The MEK1/2-inhibitor zapnometinib (PD-0184264, ATR-002; 2-(2-Chloro-4-iodophenylamino)-3,4-difluorobenzoic acid) (Figure 1), the active metabolite of CI-1040, was never developed for cancer treatment and is now used in infectious disease indications. Activation of the Raf/MEK/ERK signaling pathway is a prerequisite for various RNA viruses to ensure their propagation, while inhibition of MEK leads to viral load reduction (Planz et al., 2001; Pleschka et al., 2001; Ludwig 2009; Planz 2013; Preugschas et al., 2019; Schreiber et al., 2022). Next to this antiviral influence, MEK-inhibition modulates the innate and cellular immune response including dampening of inflammatory responses triggered by virus infections (Nakayama and Yamashita 2010; Pinto et al., 2011; Lieske et al., 2015; Schrader et al., 2018). Through this dual action, MEK inhibitors may be qualified to treat acute viral infections, where inflammation has a strong influence on the severity of the disease, like in severe influenza and COVID-19. Thus, zapnometinib was developed to treat acute viral infections, where viral propagation is depended on the activation of the Raf/MEK/ERK signaling pathway. In a recent publication, the *in vitro* and *in vivo* antiviral efficacy against influenza virus could be demonstrated, as well as the favorable pharmacokinetic profile after oral administration, when the absolute bioavailability was compared with the parental compound CI-1040 (Laure et al., 2020). Pharmacokinetics and pharmacodynamics of zapnometinib were already investigated in animal models and in humans. Here, it could be shown that MEK inhibition was maintained even after elimination of zapnometinib from plasma (Koch-Heier et al., 2022). Zapnometinib was already studied in a phase 1 clinical trial (NCT04385420) demonstrating a favorable pharmacokinetic, safety and toxicology profile. Moreover, a phase 2 clinical trial to treat hospitalized patients with severe COVID-19 was successfully executed (NCT04776044).

In the current study, we systemically investigated the pharmacokinetics, absorption, tissue distribution, metabolism and excretion of the compound following single oral doses of [^14^C]-zapnometinib (30 mg/kg; total radioactivity = 108 μCi/kg) to rats to support upcoming clinical studies in man. The present study was designed to obtain information on the time-course of plasma radioactivity concentrations, the rates, and routes of excretion of radioactivity, the extent and time-course of the distribution of radioactivity in body tissues, the metabolite profiles in plasma, urine and feces and the chemical nature of metabolites.

Maximal concentrations of radioactivity in plasma occurred at 4 h post dose in males and 2.67 h post dose in females. The terminal half-life of plasma radioactivity was 34.4 h in males and 32.2 h in females. The main component detected in plasma was unchanged zapnometinib particularly at earlier timepoints (2 and 8 h post dose). The proportion of plasma radioactivity associated with zapnometinib declined over time such that concentrations were below the limit of quantification by 168 h post dose. Trace amounts of a glucuronide conjugated and a valine conjugated metabolite of zapnometinib were detected in male plasma. Excretion of radioactivity was rapid, with >70% dose excreted within the first 24 h post dose in males and females. Radioactivity was excreted predominantly in the feces (ca 90%–93% dose) during 0–168 h post dose, mainly as unchanged compound and four major metabolites. The results of the present study provide valuable results for further clinical development and contribute to zapnometinib safety assessments in particular to its metabolite profile.

## Materials and methods

### Test material

Zapnometinib (PD-0184264, ATR-002; 2-(2-Chloro-4-iodophenylamino)-3,4-difluorobenzoic acid; Cas. No. 303175-44-2) was produced at Chemcon (Freiburg, Germany). The molecular formula is C_13_H_7_ClF_2_INO_2_ and the molecular weight is 409.56 g/mol (non-radiolabeled). ^14^C-radiolabeled zapnometinib on the chloroaniline-ring (Figure 1) was synthesized at Covance Inc. (now Labcorp Drug Development Inc., United Kingdom). The radiochemical purity was 97.5% confirmed by HPLC, the specific activity was 12.2 MBq/mg (4.98 GBq/mmol).

### Test animals and husbandry

The studies were conducted at Labcorp Early Development Laboratories Ltd. in Cambridgeshire, United Kingdom. Sprague-Dawley CD (albino) [Crl:CD (SD)] and Lister Hooded (partially pigmented) [Crl:LIS] rats used in this study were supplied by Charles River, United Kingdom. The animals were between five and 11 weeks of age at dose administration and were placed on study after 5 days of acclimation. During the acclimatization period, rats were housed in solid-bottom polycarbonate battery cages with stainless-steel lids including wood flakes grade ¾ and environmental enrichment (plastic tunnel/shelter, wooden chew block); animals were housed in groups of up to five rats per group. Prior to (for predose sample collection) and after dosing, rats assigned to the excretion phase were housed individually in glass metabolism cages specifically designed to allow the separate collection of urine and feces. At dosing, the rats weighed between 164 and 305 g (details per group see study table below). Temperature (20°C–24°C) and humidity (40%–80%) in the animal rooms were controlled acceptably throughout the studies. Rats were allowed *ad libitum* access to water from a public water supply and maintained at standard pelleted laboratory rodent diet (VRF1). Animal health was observed daily during quarantine, prior to administration and daily after dosing for any responses to treatment. The in-life experimental procedures undertaken during this study were subject to the provisions of: United Kingdom Animals (Scientific Procedures) Act 1986 Amendment Regulations 2012 (the Act).

### Study design

Seventeen male and thirteen female rats were divided into four groups: six rats for the determination of radioactivity in plasma (group 1), six rats for the determination of radioactivity in urine, feces, expired air and carcass (group 2) and eighteen rats for whole body autoradiography (group 3 and 4; see Table 1).

**TABLE 1:** Study design.

Group number	Number and sex of animals	Rat strain	No. and sex (F/M)	Age (days)/body weight (g)	Study type
1	3M + 3F	Sprague-Dawley	1M–3M, 4F–6F	54–74/249–305	Pharmacokinetics
2	3M + 3F	Sprague-Dawley	7M–9M, 10F–12F	40–60/197–215	Excretion balance
3	7M + 7F	Lister Hooded	13M–19M, 20F–26F	40–53/202–221	QWBA (pigmented)
4	4M	Sprague-Dawley	27M–30M	40–46/164–190	QWBA (albino)

M, male; F, female.

Concentrations of radioactivity were measured in plasma, blood cells and excreta by liquid scintillation analysis. Tissue concentrations in the carcass were determined using a phosphor imaging (Quantitative Whole-Body Autoradiography, QWBA) technique. Radioactivity concentrations in plasma and blood cells were also determined from pre-terminal whole blood samples and analyzed by liquid scintillation counting. Radioactive components present in plasma, urine and feces were separated and quantified using HPLC (Agilent^®^ 1260 series) with radioactivity detection and information on the chemical nature of metabolites was obtained by LC-MS/MS. All biological samples generated during this study were stored at −20 ± 10°C except during analysis, apart from blood cells which were stored at +5 ± 3°C until measurement of radioactivity was complete and then subsequently at −20 ± 10°C.

### Dose preparation and administration

Oral doses of [^14^C]-zapnometinib were administered as a suspension in 5% DMSO (v/v), 30% PEG 400 (v/v) and 7.5% (w/v) Captisol in water at a dose level of 30 mg/kg to male and female rats by gastric intubation at a dose volume of 4 ml/kg body weight. The selected dose level (30 mg/kg) was below the no observed adverse effect level (100 mg/kg), the formulation was selected based on data obtained in other nonclinical studies. The radioactive dose administered to each animal was quantified from the weight (excretion studies only) or volume of dosing formulation given, and its mean measured radioactivity concentration. The weight of test compound administered to each rat was determined from the radioactive dose administered and the specific activity of the test material.

### Pharmacokinetics

After single oral doses of [^14^C]-zapnometinib to three male and three female rats, a blood sample (ca 220 µL) was taken from each animal, *via* the jugular or tail vein at each of the following time points: 0.25, 0.5, 1, 2, 4, 8, 24, 48, 72, 96, 120 and 168 h after dose administration. All blood samples were delivered into K_2_EDTA tubes and centrifuged (ca 2000 ×g at 4°C for 10 min) to obtain plasma; blood cells were discarded. After collection of the terminal sample, rats were killed by cervical dislocation and the carcasses discarded.

### Excretion balance

Total excreta from three male and three female rats were collected after single oral doses of [^14^C]-zapnometinib. Urine was collected into containers cooled in solid CO_2_ and feces were collected separately at ambient temperature. Expired air was trapped in 1M potassium hydroxide solution. After completion of the final excreta collection, rats were killed by cervical dislocation following sedation, and the carcasses retained for analysis.

### Quantitative whole-body autoradiography

The whole-body autoradiography techniques by Ullberg and Larsson was used in this study (Ullberg and Larsson 1981). Following a single oral administration of [^14^C]-zapnometinib to seven male and seven female partially pigmented rats (Group 3) and four male albino rats (Group 4), one animal per sex (where appropriate) was killed at the following time points post dose: Group 3 (pigmented): 0.5, 2, 4, 8, 24, 72, and 168 h, Group 4 (albino): 2, 24, 72 and 168 h. At sacrifice, each rat was anaesthetized with isoflurane and a blood sample (ca 5 ml) was collected by cardiac puncture into a tube containing K_2_EDTA anticoagulant. Blood was centrifuged at 2000 ×g for 10 min at 4°C to obtain plasma, which was transferred into plain tubes pending measurement of radioactivity concentration. The blood cells were also retained for analysis.

The rat carcasses were immediately attached to a plate and frozen in a bath of n-heptane and solid CO_2_ at ca –80°C and then stored at –20 ± 10°C until taken for sectioning. Carcasses were separately processed for whole-body autoradiography/luminography. Following removal of the whiskers, legs and tail, each frozen carcass was set in a block of 2% (w/v) aqueous carboxymethylcellulose. [^14^C]-Blood standards containing six different known concentrations of radioactivity were used to construct the calibration line for the quantitative determination of tissue concentrations of radioactivity. After exposure for 3 days, the imaging plates were scanned using a FLA5000 radioluminography system. The electronic images of all sections were analyzed at Labcorp–Huntingdon using a validated image analysis package (Seescan Densitometry software, version 2.0).

### Measurement of radioactivity

Radioactivity was measured by liquid scintillation analysis using Wallac^®^ 1409 automatic liquid scintillation counters. Samples were prepared according to the company’s SOP (see Supplementary Methods). Briefly, replicate weighed samples were mixed with Ultima Gold™ scintillation cocktail (PerkinElmer, United States) for the measurement of radioactivity. Radioactivity in amounts less than twice that of the background concentration in the sample under investigation was below the limit of accurate quantification (BLQ).

### Metabolite profiling

Metabolite profiling was performed by HPLC analysis according to the company’s SOP of plasma (Group 3), urine (Group 2) and feces (Group 2) samples. Briefly, for metabolite profiling and identification, radioactivity (cpm) was measured in HPLC eluent fractions that had previously been collected at 12 s intervals for the duration of the run (65 min) into 96-well Deep Well LumaPlates and dried in a centrifugal evaporator. A Microbeta2 scintillation counter was used to count each plate, with wells simultaneously counted for 2 min, after an initial 5 min delay. A subsample of plasma was analyzed (ca 2 ml) taken from individual animals to provide representative samples, for each sex, at 2, 8 and 168 h post dose (*n* = 6). Equal proportions of the total urine weight voided from Group 2 animals was pooled to provide representative samples for each sex at 0–6 h, 6–24 h and 24–48 h post dose (*n* = 6). Equal proportions of the total feces homogenate generated from the samples voided by the three rats (Group 2) were pooled to provide representative samples for each sex at 0–24 and 24–48 h post dose (n = 4). Sample preparation was carried out due to the company’s SOPs (see Supplementary Methods). The extractability was ≥95.0% (95.0–100%) TRR (Total Radioactive Residue) for plasma and ≥88.3% (88.3%–91.3%) TRR for feces. As reference, a standard solution of zapnometinib (0.1 mg/ml in acetonitrile) was used. To assess HPLC method suitability, a column recovery was assessed on each representative sample (one per matrix). The recovery of radioactivity from the HPLC column was determined by collecting the column eluent, following an injection of a representative sample (one per matrix), and determining the radioactive content by submitting aliquots for LSC analysis. The results of these analyses were between 93.8%–105.1% column recoveries.

### Data analysis

Weight, volume, and radioactivity concentration (liquid scintillation analysis) results relating to the analysis of biological samples generated during this study were acquired and processed using DEBRA, a Laboratory Information Management System (v 5.5.4). Pharmacokinetic parameters were calculated using the computer program Phoenix™ WinNonlin version 8.3 (Pharsight Corporation, United States). Areas under the mean plasma radioactivity concentration-time curves up to the time of the last quantifiable sample (AUC_0-t_) were estimated by the linear trapezoidal rule. Areas under the mean plasma radioactivity concentration-time curves to infinite time (AUC_0-inf_) were calculated using the expression: AUC_0-inf_ = AUC_0-t_ + Ct/λz, where Ct is the last measurable concentration and λz is the elimination rate constant estimated using log-linear regression during the terminal elimination phase. Statistical evaluation was limited to basic expressions of variation such as means and standard deviation.

## Results

### Pharmacokinetics of zapnometinib in male and female rats

**FIGURE 2:**
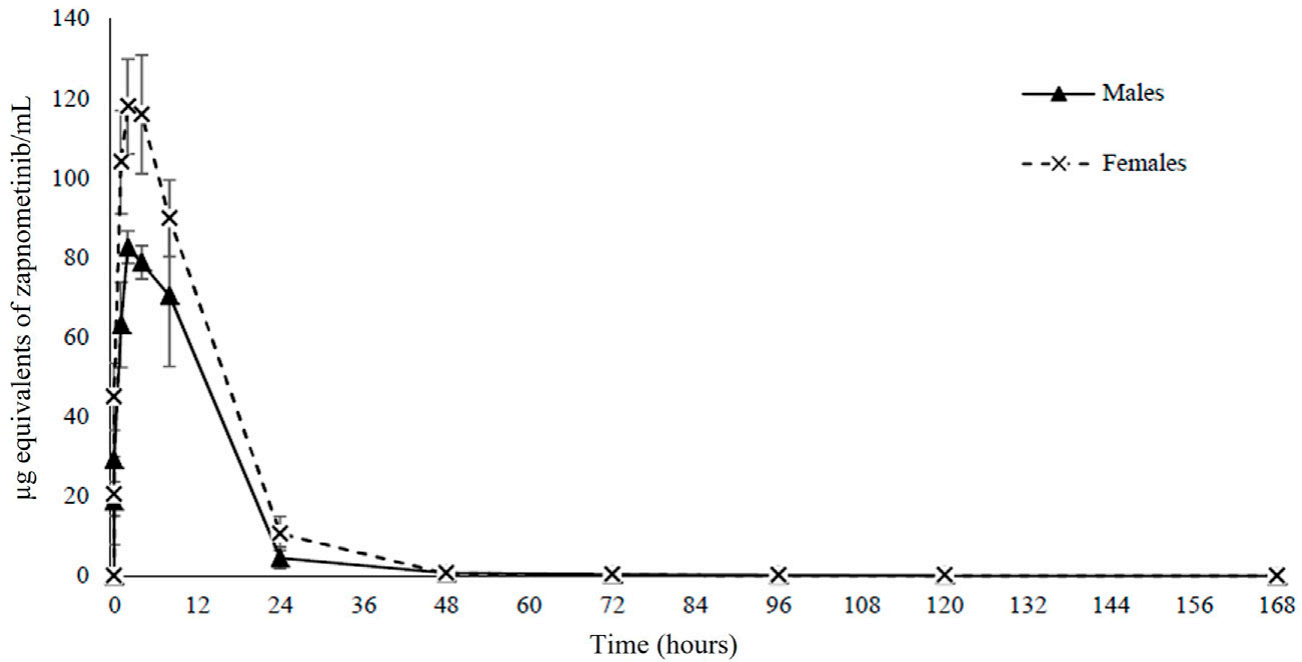
Pharmacokinetics. Mean concentrations of radioactivity in plasma for 168 h following a single oral administration of [^14^C]-zapnometinib (30 mg/kg) to male and female rats. Mean values of *n* = 3 animals are shown. Individual values see Supplementary Table S1.

To investigate a potential pharmacokinetic difference of zapnometinib between male and female rats, a single oral dose of 30 mg/kg [^14^C]-zapnometinib was administered to the male and female animals. Mean concentrations of total radioactivity in plasma were maximal at 4 h post dose in males (83.3 μg equivs/mL) and 2.67 h post dose in females (122 μg equivs/ml) (Table 2). Thereafter, mean plasma radioactivity concentrations declined in an apparent biphasic manner, reaching 0.724 μg equivs/ml in males and 0.650 μg equivs/ml in females by 48 h post dose (Figure 2). Afterwards, concentrations continued to decline and were still measurable by 168 h post dose (final sampling time) with concentrations of 0.054 μg equivs/ml in males and 0.049 μg equivs/ml in females (accounting for ca 0.06% and 0.04% of C_max_, respectively). The areas under the mean plasma radioactivity concentration-time curves up to the time of the last quantifiable sample (AUC_0-t_) of plasma radioactivity in females (1,770 μg equivs/ml) was 1.4-fold greater than in males (1,250 μg equivs/ml). The terminal half-life (t_1/2_) of total plasma radioactivity in plasma was 34.4 h and 32.2 h in males and females, respectively (Table 2). Individual pharmacokinetic parameters for female and male rats are presented in Supplementary Table S1. This data clearly demonstrated that zapnometinib is rapidly absorbed in male and female rats with only minor gender-dependent PK differences as described.

**TABLE 2:** Plasma radioactivity pharmacokinetic data following an oral dose of [^14^C]-zapnometinib (30 mg/kg) to male and female rats.

Sex	C_max_ (ng eq/ml)	T_max_ (h)	AUC_0-t_ (ng eq.h/ml)	AUC_0-inf_ (ng eq.h/ml)	t_½_ (h)
Males	83.3	4	1,250	1,260	34.4
Females	122	2.67	1,770	1,770	32.2

C_max_, maximum plasma concentration; T_max_, time of maximum plasma concentration; AUC_0-t_, area under the mean plasma radioactivity concentration-time curves up to the time of the last quantifiable sample; AUC_0-inf_, area under the mean plasma radioactivity concentration-time curves to infinite time; t_1/2_, terminal half-life. Mean values of *n* = 3 animals. Individual values see Supplementary Table S1.

### Identification of zapnometinib metabolites

**FIGURE 3:**
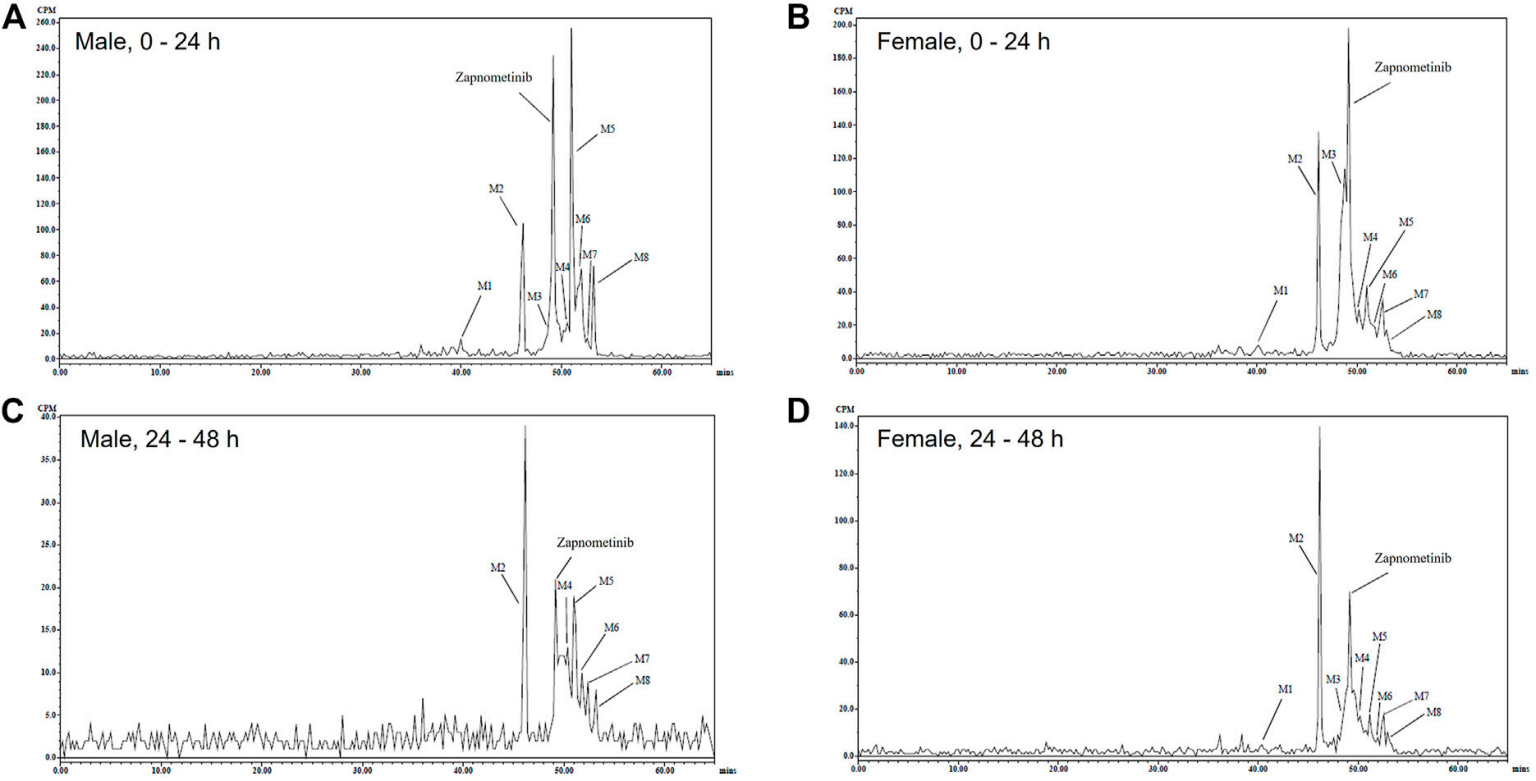
Excreted metabolites. Representative HPLC Radiochromatograms of pooled feces extracts during 0–24 **(A,B)** and 24–48 **(C,D)** h following a single oral administration of [^14^C]-zapnometinib (30 mg/kg) to male **(A,C)** and female **(B,D)** rats (Group 2).

Next, radioactive metabolic components of zapnometinib in urine, plasma and feces extracts were isolated and profiled. The components were quantified by fraction collection/scintillation analysis, as appropriate to the radioactivity concentrations. Mass spectroscopy was used to propose identities of the major circulatory and excreted metabolites, and to correlate the metabolites detected in the different matrices. Components identified by LC-MS/MS were related to the quantified HPLC analysis by comparison of related radiochromatograms and comparison of elution order. Representative HPLC radiochromatograms of pooled feces extracts collected during 0–24 and 24–48 h post dose from male and female rats (which reveal the main metabolites) were qualitatively similar with some minor sex differences (Figure 3).

Overall, eight metabolites were detected following LC-MS/MS analysis, assigned as M# based on retention times (Figure 3), although not all were present in every sample (denoted M1 to M8; see Table 3). Of the eight metabolites, three metabolites (M1–M3) were identified and were proposed as a glucuronide conjugated metabolite of zapnometinib, a hydroxylated metabolite of zapnometinib and a valine conjugated metabolite of zapnometinib. Four metabolites (M4, M5, M6 and M8) observed MS/MS fragments at m/z 408, m/z 344, m/z 282 and m/z 127, which confirmed the presence of zapnometinib (Supplementary Figure S6). Molecular formulae were proposed based on the accurate mass obtained.

**TABLE 3:** Summary of deprotonated molecular ions and characteristic fragment ions for [^14^C] Zapnometinib and identified Metabolites.

Identifier	Retention time (min)	[M-H]^-^	Proposed structure	Characteristic fragment ions (*m/z*)	Species/Matrix
M1 (glucuronic acid conjugated zapnometinib)	38.78	584	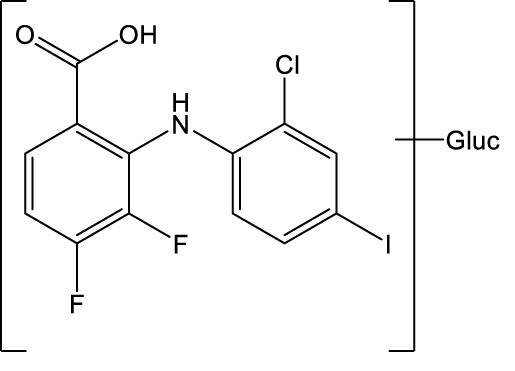	408, 344, 175, 113	RP(m)^a^, RU(f), RF(m)
M2 (hydroxylated zapnometinib)	44.56	424	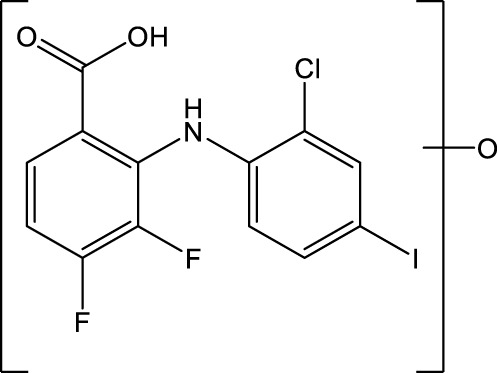	360, 340, 127	RU(f)^a^, RF(m)
M3 (valine conjugated zapnometinib)	46.97	507	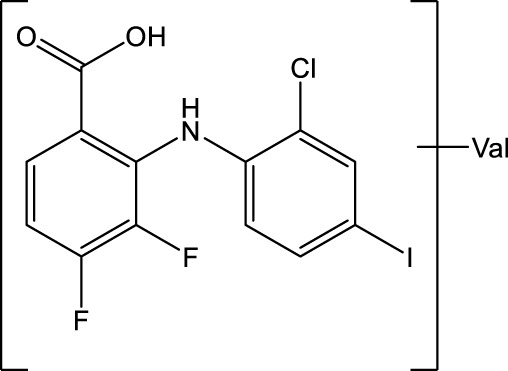	463, 427, 344, 127	RP(m)^a^, RF(m)
Zapnometinib	47.42	408	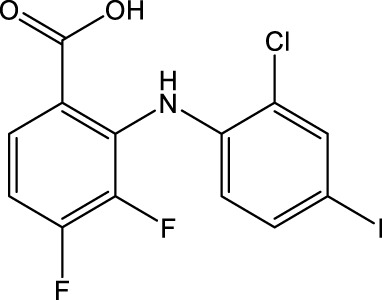	364, 344, 127	RP(m), RU(f), RF(m)
M4	48.65	853	Not identified	364, 344, 282, 127	RF(m)
M5	49.18	853	Not identified	364, 344, 282, 127	RF(m)
M6^b^	49.77	851	Not identified	364, 344, 282, 127	RF(m)
M7	50.40^b^	Not identified	Not identified	Not applicable	RF(m)
M8	51.19	839	Not identified	364, 344, 282, 127	RF(m)

^a^ Peak observed in MS but not in radioanalysis.

^b^ Radiochromatographic retention time obtained from a Group 2 male 0–24 h feces pool extract.

Mass chromatographic retention times obtained from a Group 2 male 0–24 h feces pool extract.

RP(m) Group 3 male Rat 2 h plasma pool extract; RU(f) Group 2 Female Rat 6–24 h urine pool.

RF(m) Group 2 male Rat 0–24 h Feces pool extract.

Four metabolites (M4, M5, M6 and M8) were reported with corresponding *m/z* ([M-H-]) values and possible molecular formulae. Metabolite M7 was not identified by LC-MS (Table 3).

In urine the metabolic profiles at each time point were qualitatively similar, though minor sex differences were observed. Up to 29 radioactive components were detected although not all were present in every sample. The hydroxylated metabolite of zapnometinib (M2) was also detected in female urine by mass spectrometry and the glucuronide conjugated metabolite (M1) in both males and females though only in trace amounts. A summary of the amounts (% dose) of the principal components separated by HPLC in pooled urine is presented in Supplementary Table S2 (male rats) and Supplementary Table S3 (female rats).

The main fecal metabolites in male rats were attributable to M2, M5 and M6 (each individually accounting for ca 12–20% dose during 0–48 h, see Supplementary Table S4), though the identity of M5 and M6 was not confirmed by LC-MS/MS analysis, possible deprotonated molecular ions were proposed (Table 3). In female rats, the main fecal metabolites were attributable to M2 and M3 (proposed as a hydroxylated and a valine conjugated metabolite of zapnometinib) each individually accounting for ca 15%–19% dose during 0–48 h (Supplementary Table S5). During 0–24 h, the major peak was unchanged zapnometinib with a retention time of 47.42 min followed by metabolite M2, accounting for 12.3% sample radioactivity (9.76% dose) in male (Figure 3A) and 13.6% sample radioactivity (9.32% dose) in female rats (Figure 3B), as well as M5 (proposed as an isomer of M4 with proposed deprotonated molecular formulae of either C_39_H_45_O_7_N_2_ClF_2_I or C_36_H_49_O_7_N_2_ClF_2_IS) accounting for 23.1% sample radioactivity (18.33% dose) in male rats, but only <6% sample radioactivity in female rats. During 24–48 h post dose, the major peak detected was hydroxylated zapnometinib (M2), still followed by unchanged zapnometinib which accounted for 21.5% sample radioactivity (2.15% dose) in male (Figure 3C) and 28.5% sample radioactivity (6.30% dose) in female rats (Figure 3D). The proportion of unchanged zapnometinib slightly decreased with time. In summary, the most abundant metabolites of zapnometinib detected in feces 0–48 h post dose were hydroxylated and valine-conjugated compound in female rats and hydroxylated compound and possible deprotonated isomers of zapnometinib in male rats, but unchanged substance remained the main component.

The metabolic profiles of plasma extracts at each time point (2, 8 and 168 h post dose) were qualitatively similar and no overt sex differences were observed. The glucuronide conjugated and valine conjugated metabolites of zapnometinib (M1 and M3) were also detected in male plasma by mass spectrometry, though only in trace amounts. At 2 and 8 h post dose unchanged zapnometinib was the major component of plasma radioactivity and the proportions and concentrations of zapnometinib were relatively similar between males and females [Supplementary Table S6 (male) and Supplementary Table S7 (female)]. Unidentified components P1–P4 were also detected as minor components (Supplementary Figure S4).

These data clearly demonstrate that the main component detectable in plasma 0–168 h post dose is the unchanged, active compound zapnometinib, which confirms its good bioavailability. Urinary excretion was a minor route of elimination (ca 3% and 4% dose in males and females, respectively; Table 4) and no single metabolite attributable to >1% dose was detected (Supplementary Figure S5).

**TABLE 4:** Excretion of radioactivity during 0–168 h after administration.

Sample	% Administered dose	
	Male	Female
Urine	2.83	4.12
Feces	90.45	93.32
Cage wash	0.22	0.60
Expired air	0.02	0.02
Carcass	0.20	0.19
Total recovery	93.72	98.26

### Excretion balance of [^14^C]-zapnometinib

**FIGURE 4:**
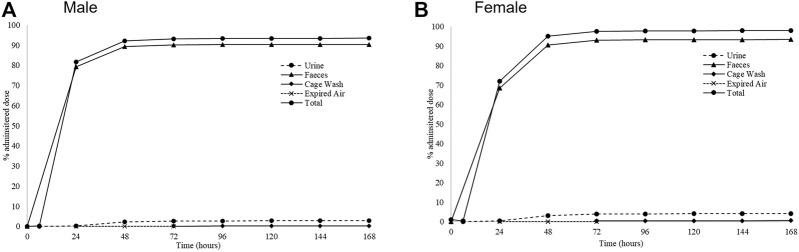
Excretion routes of zapnometinib. Mean cumulative excretion of radioactivity during 168 h following a single oral administration of [^14^C]-zapnometinib (30 mg/kg) to male **(A)** and female **(B)** rats (Group 2). Mean values of *n* = 3 animals are shown. Individual values and SD see Supplementary Tables S2, S3.

Cumulative rates of excretion following a single oral administration of [^14^C]-zapnometinib to male and female rats showed a mean total recovery of [^14^C]-zapnometinib of 93.72% in males and 98.26% in females during the 168-h sample collection period. Only a small proportion of the administered radioactivity was detected in the carcasses (means of 0.20% and 0.19% in males and females, respectively) at 168 h post dose, indicating that elimination was essentially complete by the end of the sample collection period. Radioactivity was excreted predominantly in the feces with means of 90.45% (males) and 93.32% (females) of administered dose excreted by this route during 0–168 h post dose. Urinary excretion was a minor route of elimination accounting for mean totals of 2.83% and 4.12% dose, with cage washings accounting for a further 0.22% and 0.60% dose in males and females, respectively (Figure 4; Table 4). Excretion of radioactivity was rapid with mean totals of ca 82% and 72% dose recovered in urine, feces and cage washings by 24 h post dose in males and females, respectively (Supplementary Tables S8, S9). There was very little radioactivity detected in the expired air of either male or female animals (a mean total of 0.02% dose), indicating that [^14^C]-zapnometinib was radiolabelled in a metabolically stable position. The results indicate no substantial sex differences in the metabolic excretion patterns; furthermore, the elimination of radioactivity was essentially complete at 168 h post dose.

### Distribution of zapnometinib to different tissues

**FIGURE 5:**
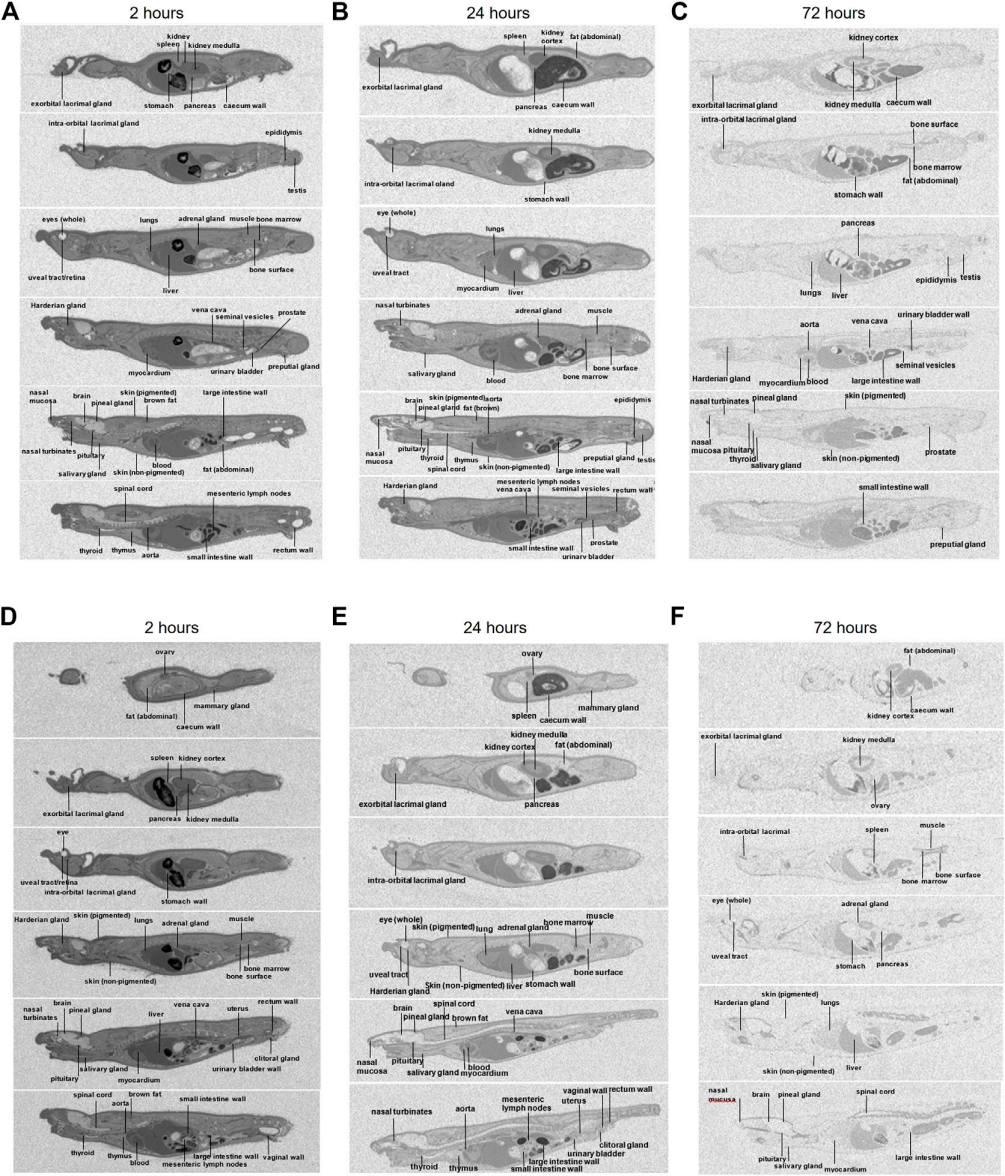
Whole body autoradiography. Distribution of radioactivity at 2, 24 and 72 h following a single oral administration of [^14^C]-zapnometinib (30 mg/kg) to male **(A–C)** and female **(D–F)** partially pigmented rats (Group 3). Whole body autoradiogram for one representative animal is shown.

Whole-body radioluminograms for one representative animal were performed to investigate the distribution of 30 mg/kg [^14^C]-zapnometinib following a single oral administration to male and female pigmented rats. The distribution of radioactivity in male and female rats at 2, 24 and 72 h post dose is shown in Figure 5. Radioactivity was rapidly and widely distributed with measurable concentrations observed in all tissues at 0.5 h post dose (first sampling time) (Table 5, 6). At 2 h, radioactivity concentrations increased in all tissues in male rats except for the whole eye (0.767 μg equivs/g), thyroid (21.3 μg equivs/g) and large intestine wall (5.85 μg equivs/g). Maximum concentrations were observed in the kidney medulla (108 μg equivs/g), pancreas (46.6 μg equivs/g) and bone surface (30.3 μg equivs/g). Greatest radioactivity concentrations were present in the cardiac blood (112 μg equivs/g), kidney medulla (108 μg equivs/g), kidney cortex (94.8 μg equivs/g), liver (81.3 μg equivs/g) and lungs (77.3 μg equivs/g). All remaining tissues contained ≤70.0 μg equivs/g. Lowest concentrations were present in the seminal vesicles (5.54 μg equivs/g), spinal cord (2.49 μg equivs/g), brain (1.79 μg equivs/g) and whole eye (0.767 μg equivs/g) (Table 5). In female rats, radioactivity increased in approximately half of all tissues of which maximum concentrations were observed in blood cells, aorta, liver, adrenal glands, exorbital lacrimal gland, pituitary gland, non-pigmented skin and caecum wall. Greatest concentrations were observed in cardiac blood (145 µg equivs/g), liver (127 µg equivs/g), lungs (114 µg equivs/g), uterus (96.6 µg equivs/g), plasma (80.1 µg equivs/g), aorta (75.9 µg equivs/g) and myocardium (75.0 µg equivs/g). All remaining tissues contained ≤74.4 µg equivs/g. Lowest measurable concentrations were present in the abdominal fat (7.69 µg equivs/g), mesenteric lymph nodes (6.95 µg equivs/g), spinal cord (3.84 µg equivs/g), brain (3.48 µg equivs/g) and whole eye (1.17 µg equivs/g) (Table 6). Maximal concentrations of radioactivity were generally observed at 8 h post dose in males and between 0.5–8 h post dose in females. Thereafter, concentrations significantly declined in all tissues such that they were approaching or below the limit of quantification at 168 h post dose in males and females, respectively (Tables 5, 6).

**TABLE 5:** Concentrations of radioactivity in tissues determined by QWBA following a single oral administration of [^14^C] zapnometinib (30 mg/kg) to male partially pigmented rats (Group 3).

Tissue type	Tissue/organ	Concentrations of total radioactivity (µg equivalents zapnometinib/g)						
		13M 0.5 h	14M 2 h	15M 4 h	16M 8 h	17M 24 h	18M 72 h	19M 168 h
Non QWBA	Plasma^a^	47.7	70.0	75.0	**77.9**	20.1	0.661	0.062
	Blood cells^b^	5.78	11.3	**14.5**	13.0	2.89	0.105	0.036
Circulatory	Aorta	35.6	69.7	43.0	**209**	25.8	0.384	BLQ
	Blood (cardiac)	49.5	112	169	**335**	38.7	0.856	BLQ
	Vena cava	24.2	61.7	50.5	**198**	13.5	0.308	BLQ
Nervous	Brain	1.09	1.79	3.21	**6.79**	0.637	BLQ	BLQ
	Spinal cord	1.20	2.49	2.66	**7.52**	0.439	BLQ	BLQ
Ocular	Eye (whole)	1.05	0.767	**2.27**	1.60	0.460	BLQ	BLQ
	Uveal tract/retina	5.05	29.1	60.5	**87.9**	11.5	BLQ	BLQ
Visceral	Kidney cortex	30.6	94.8	**115**	89.3	21.6	0.959	0.244
	Kidney medulla	32.7	**108**	67.0	76.8	7.52	0.313	0.174
	Liver	69.4	81.3	112	**202**	18.8	0.752	0.398
	Lungs	47.1	77.3	85.5	**111**	14.1	0.286	BLQ
	Myocardium	19.6	49.8	67.5	**123**	10.4	0.281	BLQ
	Spleen	11.6	21.8	**28.4**	26.4	3.07	BLQ	BLQ
Glandular/Secretory	Adrenal gland	31.5	57.6	95.5	**112**	7.20	BLQ	BLQ
	Exorbital lacrimal gland	8.69	50.3	**78.8**	49.5	7.50	0.232	BLQ
	Harderian gland	3.71	38.0	51.9	**116**	10.7	0.330	0.178
	Intra-orbital lacrimal gland	20.4	67.2	60.5	**94.5**	7.33	0.185	0.265
	Mesenteric lymph nodes	12.6	33.7	14.3	**37.4**	3.14	BLQ	BLQ
	Nasal mucosa	1.68	21.4	21.6	**22.4**	2.35	BLQ	BLQ
	Nasal turbinates	3.64	16.8	24.3	**68.2**	2.94	0.135	0.311
	Pancreas	23.5	**46.6**	44.4	35.7	6.00	0.149	BLQ
	Pineal gland	18.2	23.1	45.6	**89.9**	7.31	0.146	BLQ
	Pituitary	19.5	30.2	41.9	**80.9**	12.5	0.179	BLQ
	Salivary gland	18.1	29.2	44.2	**107**	7.04	0.161	BLQ
	Thymus	3.51	8.41	16.3	**33.9**	3.03	BLQ	BLQ
	Thyroid	24.2	21.3	51.8	**82.4**	9.37	0.310	BLQ
Musculo-skeletal	Bone marrow	12.9	22.9	**56.4**	49.3	5.38	0.152	BLQ
	Bone surface^c^	9.63	**30.3**	27.8	24.9	8.53	1.04	BLQ
	Fat (abdominal)^c^	3.71	7.26	7.93	**9.10**	1.83	0.169	BLQ
	Fat (brown)	16.6	41.3	52.4	**101**	5.53	BLQ	BLQ
	Muscle	12.9	21.0	27.8	**38.4**	2.53	BLQ	BLQ
	Skin (non-pigmented)	2.71	26.4	43.7	**85.5**	7.03	0.210	BLQ
	Skin (pigmented)	3.54	15.2	32.5	**60.6**	13.2	0.436	BLQ
Reproductive	Epididymis	5.21	22.3	**42.8**	42.4	4.31	0.193	BLQ
	Preputial gland	12.0	29.2	**82.0**	57.1	5.62	0.250	BLQ
	Prostate	6.96	22.7	42.1	**54.8**	12.4	0.119	BLQ
	Seminal vesicles	0.803	5.54	13.9	**20.8**	8.58	0.146	BLQ
	Testis	2.28	21.3	25.8	**51.2**	5.43	0.164	BLQ
Excretory	Caecum wall	6.13	30.7	**63.7**	43.5	8.65	0.466	0.133
	Large intestine wall	10.0	5.85	38.4	**110**	7.77	0.153	BLQ
	Rectum wall	8.89	19.2	40.6	**248**	11.7	BLQ	BLQ
	Small intestine wall	28.2	27.1	49.3	**64.7**	9.70	0.244	BLQ
	Stomach wall	12.7	28.0	51.3	**63.6**	3.61	0.178	0.121
	Urinary bladder wall	3.11	12.5	15.2	**115**	7.16	1.80	BLQ

^a^ Determined by direct liquid scintillation analysis (LLOQ = 0.007–0.013 µg equiv zapnometinib/g).

^b^ Determined by oxidation and liquid scintillation analysis (LLOQ = 0.011–0.013 µg equiv zapnometinib/g).

^c^ Tissue corrected for quenching.

For all QWBA measurements: Values in bold represent maximum tissue concentrations (T_max_). Upper and lower limits of quantification = 510 and 0.118 µg equiv zapnometinib/g of tissue, respectively

QWBA, Quantitative whole-body autoradiography; BLQ, Tissue radioactivity concentration below the lower limit of quantification (LLOQ).

**TABLE 6:** Concentrations of radioactivity in tissues determined by QWBA following a single oral administration of [^14^C] zapnometinib (30 mg/kg) to female partially pigmented rats (Group 3).

Tissue type	Tissue/organ	Concentrations of total radioactivity (µg equivalents zapnometinib/g)						
		20F 0.5 h	21F 2 h	22F 4 h	23F 8 h	24F 24 h	25F 72 h	26F 168 h
Non QWBA	Plasma^a^	66.7	80.1	**91.0**	73.5	7.09	0.399	0.124
	Blood cells^b^	9.12	**14.0**	13.2	12.5	1.01	0.108	0.053
Circulatory	Aorta	71.4	**75.9**	65.3	73.9	2.96	0.421	BLQ
	Blood (cardiac)	**209**	145	158	145	12.9	0.741	BLQ
	Vena cava	41.4	63.0	**83.0**	75.1	4.73	BLQ	BLQ
Nervous	Brain	4.25	3.48	**5.43**	3.53	0.366	0.195	BLQ
	Spinal cord	4.19	3.84	**6.17**	3.21	0.360	0.175	BLQ
Ocular	Eye (whole)	0.500	1.17	0.929	**2.35**	0.337	0.276	BLQ
	Uveal tract/retina	24.7	38.4	64.8	**75.1**	3.03	0.484	BLQ
Visceral	Kidney cortex	88.3	74.0	**95.9**	90.1	9.29	1.56	0.541
	Kidney medulla	77.3	62.2	**79.3**	71.7	4.59	0.430	0.211
	Liver	83.7	**127**	112	106	9.11	1.57	1.12
	Lungs	114	114	**121**	67.7	5.47	0.413	BLQ
	Myocardium	**87.9**	75.0	85.9	58.5	4.00	0.319	BLQ
	Spleen	**37.2**	24.2	28.9	22.0	1.56	0.320	0.181
Glandular/Secretory	Adrenal gland	53.7	**74.4**	57.5	57.7	3.13	0.364	0.204
	Exorbital lacrimal gland	46.7	**52.7**	49.1	47.2	3.21	0.559	BLQ
	Harderian gland	18.2	50.4	**75.1**	50.9	2.54	0.513	0.251
	Intra-orbital lacrimal gland	30.9	38.2	**85.8**	63.0	2.35	0.318	0.304
	Mesenteric lymph nodes	**37.0**	6.95	11.1	15.8	1.06	0.153	NS
	Nasal mucosa	**23.9**	NS	8.53	NS	2.30	0.271	BLQ
	Nasal turbinates	13.2	16.5	**34.1**	22.0	2.55	0.362	0.293
	Pancreas	**56.5**	52.7	44.7	38.5	2.38	0.406	BLQ
	Pineal gland	39.2	44.1	**53.9**	38.7	2.92	0.447	BLQ
	Pituitary	62.2	**62.9**	57.7	47.1	3.98	0.272	BLQ
	Salivary gland	**64.4**	61.9	51.9	41.9	2.95	0.312	BLQ
	Thymus	17.0	18.7	**22.4**	16.8	0.888	0.336	BLQ
	Thyroid	**60.7**	55.6	52.5	55.6	3.31	0.382	BLQ
Musculo-skeletal	Bone marrow	35.6	31.7	**43.1**	39.2	3.87	0.665	0.423
	Bone surface^c^	19.5	22.8	26.8	**36.3**	7.03	9.30	6.15
	Fat (abdominal)^c^	3.86	7.69	**11.2**	10.5	0.839	0.294	BLQ
	Fat (brown)	33.7	41.4	36.3	**43.6**	2.33	0.424	BLQ
	Muscle	23.7	20.4	**23.8**	23.2	0.792	0.210	BLQ
	Skin (non-pigmented)	13.3	**41.1**	33.7	22.9	4.25	0.513	BLQ
	Skin (pigmented)	11.5	42.1	**83.2**	45.8	3.04	0.504	BLQ
Reproductive	Clitoral gland	9.88	37.1	**137**	37.9	2.55	BLQ	BLQ
	Mammary gland	21.3	19.1	21.6	**40.7**	1.79	BLQ	BLQ
	Ovary	77.0	58.6	**108**	92.4	4.65	0.511	0.846
	Uterus	49.7	96.6	**96.1**	92.2	2.53	0.575	BLQ
	Vaginal wall	20.4	43.1	44.1	**56.0**	3.47	0.289	BLQ
Excretory	Caecum wall	34.0	**64.0**	58.0	35.4	6.27	0.468	0.527
	Large intestine wall	21.6	33.7	43.9	**79.7**	3.06	0.409	0.550
	Rectum wall	10.8	47.7	**58.1**	39.0	4.63	0.348	0.805
	Small intestine wall	53.8	46.3	**60.8**	43.6	2.10	0.322	BLQ
	Stomach wall	50.3	45.4	**60.9**	46.0	1.99	0.356	0.370
	Urinary bladder wall	33.0	28.2	35.8	**52.1**	3.57	NS	BLQ

^a^ Determined by direct liquid scintillation analysis (LLOQ = 0.009–0.019 µg equiv zapnometinib/g).

^b^ Determined by oxidation and liquid scintillation analysis (LLOQ = 0.011–0.031 µg equiv zapnometinib/g).

^c^ Tissue corrected for quenching.

For all QWBA measurements: Values in bold represent maximum tissue concentrations (T_max_). Upper and lower limits of quantification = 510 and 0.118 µg equiv zapnometinib/g of tissue, respectively.

QWBA, Quantitative whole-body autoradiography; BLQ, Tissue radioactivity concentration below the lower limit of quantification (LLOQ); NS, Tissue not present on sections.

At 24 h, concentrations of radioactivity significantly declined in all tissues though the pattern of distribution remained largely unchanged (except for the bone surface in females) with greatest concentrations observed in the cardiac blood, aorta, kidney cortex, plasma, liver, lungs and vena cava (13.5–38.7 μg equivs/g) in male and in cardiac blood, kidney cortex, liver, plasma and bone surface in female rats (12.08–7.03 μg equivs/g). Lowest concentrations were generally observed in the nervous and ocular tissues, in female rats also in muscle and abdominal fat (Figure 5; Tables 5, 6).

At 72 h post dose, concentrations of radioactivity in most tissues continued to decline with approximately 25% of tissues being below the limit of quantification (with exception of the bone surface in female rats). Greatest measurable concentrations were observed in the urinary bladder wall (in male; Table 5), bone surface, kidney cortex, cardiac blood and liver (1.80–0.752 μg equivs/g) and additionally 0.665–9.30. 752 μg equivs/g in the bone marrow in female rats (Table 6).

To be able to assess possible melanin binding of zapnometinib, non-pigmented (albino) rats were additionally investigated. Following a single oral administration of 30 mg/kg [^14^C]-zapnometinib to male albino rats, radioactivity was rapidly and widely distributed with maximal concentrations observed in all tissues at 2 h post dose (first sampling time) (Supplementary Figure S1; Supplementary Table S10). Thereafter concentrations generally declined such that they were approaching or below the limit of quantification in the majority of tissues by 72 h post dose. Where measurable, greatest concentrations were generally observed in the blood (cardiac), intra-orbital lacrimal gland, kidney cortex, aorta, liver and lungs. Lowest concentrations were generally observed in the nervous tissues, ocular tissues and blood cells. Where measurable, concentrations in tissues were generally lower than those in plasma. During 2–24 h post dose, concentrations of radioactivity in tissues were generally less than the corresponding plasma concentrations.

In summary, the distribution pattern was generally similar in males and females, with greatest concentrations generally observed in the circulatory (particularly cardiac blood) and visceral tissues. Overall, the tissue distribution in male albino rats was comparable to the results in male pigmented rats.

## Discussion

Zapnometinib is an orally available MEK-inhibitor with a dual therapeutic effect that is currently under development for the treatment of severe viral diseases like COVID-19 and influenza. Preclinical *in vitro* and *in vivo* studies demonstrated efficacy and safety of the drug (Laure et al., 2020; Schreiber et al., 2022; Koch-Heier et al., 2022), which allowed further investigations in Phase 1 (NCT04385420) and Phase 2 (NCT04776044) clinical trials in humans. Zapnometinib is the active metabolite of CI-1040, a drug that was developed to treat cancer (LoRusso et al., 2005, Rinehart et al., 2004; Barrett et al., 2008; Seibold-Leopold et al. 1999). In contrast to CI-1040, zapnometinib never was developed for cancer treatment.

Orally administered [^14^C]-zapnometinib was rapidly and widely distributed, indicating that diffusion across cell membranes seems not to be a rate-determining factor. The pattern of distribution was generally similar in males and females, with greatest concentrations generally observed in the circulatory (particularly cardiac blood) and visceral tissues, though notable radioactive concentrations were observed in the female reproductive organs. Lowest concentrations were generally observed in the whole eye, blood cells, thymus, abdominal fat and nervous tissues.

Maximal concentrations of radioactivity in plasma occurred between 2.67 and 4 h post dose with a terminal half-life of plasma radioactivity between 32.2 and 34.4 h. The systemic exposure (C_max_ and AUCt) to total plasma radioactivity of female rats was 1.4-fold (AUCt) and 1.5-fold (C_max_) higher than that of males. Radioactive concentrations were detectable up to 168 h post dose (final time point) in males and females, respectively. These preclinical pharmacokinetic data are important for clinical trial development, in particular for estimation of the dose rationale.

During 0.5–72 h post dose in males and females, concentrations of radioactivity in tissues were generally less than the corresponding plasma concentrations except for tissues such as the aorta (particularly in males), blood (cardiac), kidney cortex, liver, lungs, ovary and uterus. Concentrations of zapnometinib in plasma and in lungs were comparable in male and female rats, even though marginal increased in female rats between 0.5–4 h. For treatment of acute viral lung infections like influenza and COVID-19, deposition of zapnometinib in the lung must be assured and could be confirmed in this study.

Melanin binding affects drug distribution and retention in pigmented ocular tissues, thereby affecting drug response, duration of activity and toxicity (Rimpela et al., 2018). In the present study concentrations of radioactivity were below the limit of quantification in pigmented skin and ocular tissues at 168 h post dose, suggesting limited association of drug-related material binding to melanin. For further confirmation, the affinity of zapnometinib for melanin was evaluated by comparing tissue distribution in albino rats. Here, tissue distribution in male albino rats was like male pigmented rats, which further supports the hypothesis that the association of zapnometinib or related material with melanin is limited.

In a plasma protein binding assay in human, dog and rat plasma, zapnometinib showed high protein binding, and a very low unbound fraction (data not shown). Nevertheless, the compound shows good efficacy when PK and PD were correlated (Koch-Heier et al., 2022). A possible interaction with albumin, serving as a drug-carrier and reservoir for the molecule, is further investigated. In this study, the extractability of plasma radioactivity was >90% TRR (total radioactive residue) at all measured time points, indicating that there was negligible permanent binding of radioactivity to plasma proteins.

[^14^C]-zapnometinib was rapidly absorbed in rats, but metabolization was rather slow. This contrasts with CI-1040, which underwent extensive oxidative metabolism and glucuronidation in animals and humans (Wabnitz et al., 2004). The herein described metabolites were mainly detected in feces and only in trace amounts in plasma, where MEK inhibition is necessary for drug efficacy. In plasma, the active, unchanged compound was predominantly abundant 0–168 h after administration, which ensures drug efficacy at the desired locations. A glucuronide conjugated metabolite of zapnometinib and a valine conjugated metabolite of zapnometinib (M1 and M3) could also be detected in male plasma, though only in trace amounts. The main fecal metabolites were attributable to hydroxylated, valine-conjugated or possible deprotonated compound. No single metabolite attributable to >1% dose was detected in collected urine.

During drug metabolism, conjugation with glucuronic acid or amino acids leads to more polar compounds, which facilitates elimination of drugs from the body (Phang-Lyn and Llerena, 2022). For CI-1040 fourteen metabolites were characterized. Many of the hydroxylated metabolites were subsequently glucuronidated leading to a fast excretion in the feces (Wabnitz et al., 2004). Since glucuronidation was not observed to such an extend with zapnometinib in rats, this could be a reason for a prolonged presence of zapnometinib in animals and humans compared to CI-1040 (Lorusso et al., 2005; Koch-Heier et al., 2022). In summary, the metabolic profile confirms the good stability and bioavailability with normal metabolization of the compound for elimination of the drug from the body, demonstrating an important role for metabolizing enzymes for the ADME profile of zapnometinib, and no distinctive features that would speak against good compatibility and safety of zapnometinib.

Another good feature is the rapid excretion of the compound after zapnometinib treatment, with ca 82% and 72% dose excreted within the first 24 h post dose in males and females, respectively. There were no substantial sex differences between patterns of excretion, and the elimination of radioactivity was essentially complete at 168 h post dose only a small proportion of the administered radioactivity was detected in the carcasses (means of ca 0.2%) at 168 h post dose. Radioactivity was excreted predominantly in the feces with means of ca 90% of dose excreted by this route during 0–168 h post dose. Urinary excretion was a minor route of elimination (3–4% dose). This is in line with observations from the parental compound CI-1040. Renal elimination of [^14^C]-CI-1040 for rat and monkey was minimal (<5% of dose). Wabnitz and colleagues also analyzed urine from a cancer patient which showed virtually no metabolites of CI-1040 to be present. Thus, it is suspected that most of zapnometinib, like CI-1040, would be excreted in the bile in animals and humans (Wabnitz et al., 2004).

**FIGURE 6:**
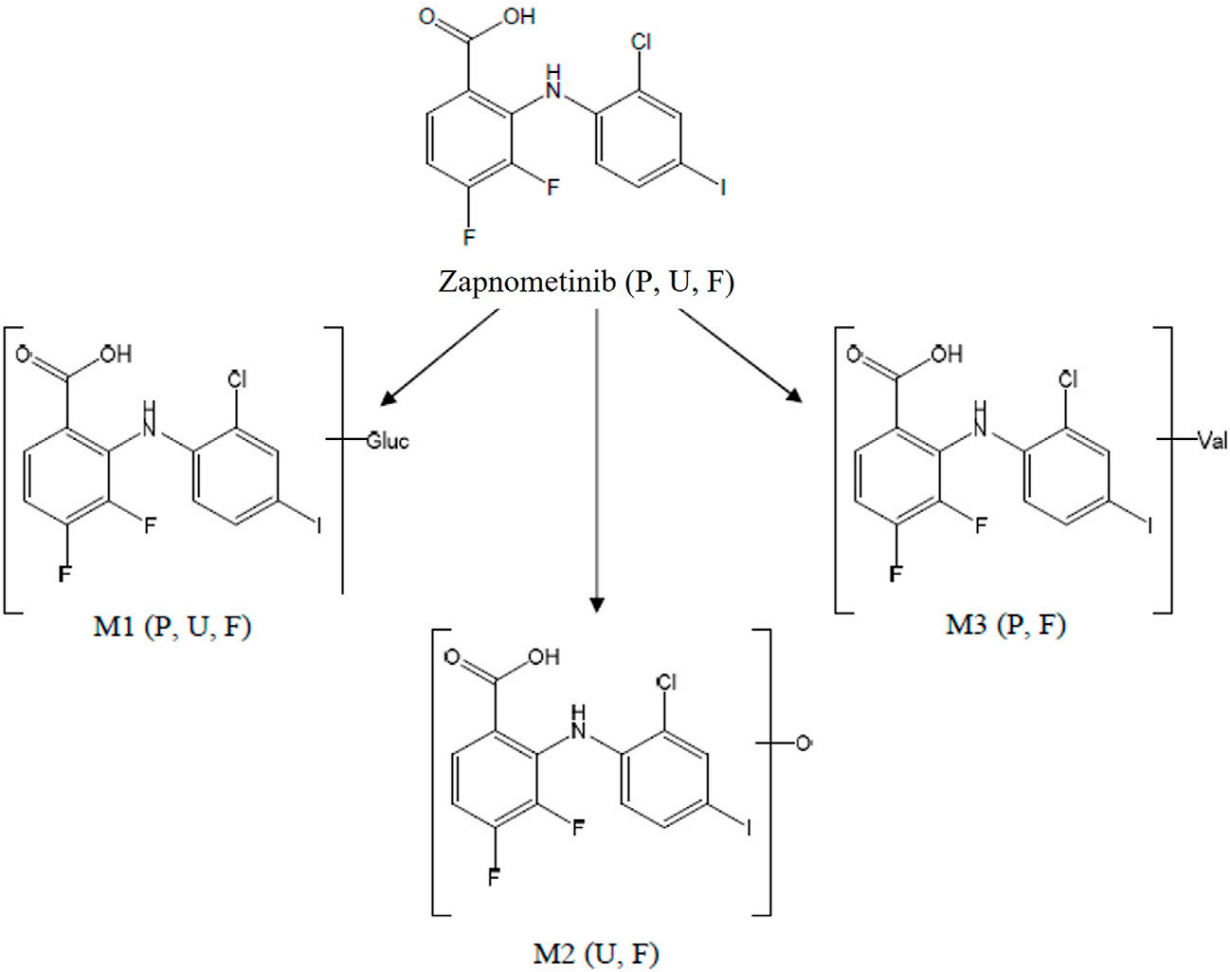
Proposed biotransformation pathway of zapnometinib. Metabolites (M1–M3) identified *via* mass spectrometry in feces (F), plasma (P) or urine (U) in male and female rats following a single oral administration of [^14^C]-zapnometinib (30 mg/kg), separated and quantified by HPLC with radioactivity detection.

In conclusion, zapnometinib was rapidly absorbed and widely distributed in male and female rats. Unchanged zapnometinib was the main component detected in plasma particularly at earlier timepoints. Metabolism of zapnometinib included a hydroxylated, a glucuronide conjugated and a valine conjugated metabolite of zapnometinib (Figure 6) and possible deprotonated isomers. Zapnometinib was predominantly and rapidly excreted in the feces as unchanged zapnometinib, though four main metabolites, each individually accounting for >10% administered dose, were detected. Urinary excretion was a minor route of elimination and no metabolite attributable to >1% dose was detected. Comparable low concentrations of zapnometinib were detected in pigmented and albino skin, suggesting limited association of drug-related material binding to melanin. In summary, the good absorption, favorable distribution, very limited metabolism and complete and rapid excretion makes zapnometinib a valuable tool for the desired indications in and outside infectious disease. Furthermore, the findings from this study will contribute to the understanding of the pre-clinical pharmacology of zapnometinib and follow-up compounds thereof.

## Data Availability

The original contributions presented in the study are included in the article/Supplementary Material, further inquiries can be directed to the corresponding author.
