# Supplementary material for: Pharmacokinetics, absorption, distribution, metabolism and excretion of the MEK inhibitor zapnometinib in rats
Source: Front Pharmacol. 2022 Dec 5;13:1050193. doi: 10.3389/fphar.2022.1050193 (PMC9760738; doi:10.3389/fphar.2022.1050193)
Supplement: Supplementary file 1 [file DataSheet1.pdf]

Supplementary for:

Pharmacokinetics, Absorption, Distribution, Metabolism and Excretion of the MEK Inhibitor Zapnometinib in Rats

Yvonne Füll, Christian Wallasch, Ashley Hilton and Oliver Planz

Supplementary Methods

Supplementary Figures S1 - S6

Supplementary Tables S1 - S10

Supplementary methods:

Sample preparation

For HPLC analysis, a portion of each plasma sample (ca 2 mL; 1 volume) was extracted with a single aliquot (3 volumes) of acetonitrile/methanol 4:1 (v/v). After addition of solvent, the samples were vortex mixed (ca 1 minute), sonicated (ca 10 minutes), then centrifuged (2000 × g for 10 minutes at 4°C) and the supernatant transferred to a clean tube. The resulting residue was extracted a further two times with the following solvents following the same procedure: Extract 2 – Acetonitrile/Methanol 4:1 (v/v); Extract 3 – Acetonitrile/Methanol 1:1 (v/v).

Each extract was weighed and duplicate weighed aliquots (0.1 mL) taken for measurement of radioactivity in order to calculate the extraction efficiency. The extracts were combined and concentrated under a constant stream of nitrogen gas to near dryness and reconstituted in acetonitrile/0.1% aqueous formic acid, 1:1 (v/v) by vortex mixing, sonication and centrifugation at 10000 × g for 5 min at 4°C. The concentrates were weighed, and duplicate weighed aliquots (0.025 – 0.05 mL) taken for measurement of radioactivity to calculate recovery following concentration. An aliquot of each concentrated extract was injected into the HPLC.

A portion of each pooled urine sample was centrifuged at 10000 × g for 5 minutes at 4°C to remove any particulate material and then injected directly into the HPLC. Feces homogenate samples were allowed to thaw and the total remaining sample was transferred to pre-weighed plastic centrifuge pots. After measurement of total sample weight, each sample was mixed with acetonitrile/methanol 4:1 (v/v) (nominally 3 × sample weight) using a sample shaker (ca 1 minute). Following sonication (ca 10 minutes) and centrifugation (2000 × g for 10 minutes), the supernatant was decanted and weighed. The resulting residue was extracted a further two

times with the following solvents following the same procedure: Extract 2 – Acetonitrile/methanol 4:1 (v/v); Extract 3 – Acetonitrile/Water 1:1 (v/v). Duplicate weighed aliquots of each supernatant (ca 0.1 mL) were mixed directly with Ultima Gold scintillation cocktail for measurement of radioactivity concentrations. The extracted residue was allowed to air dry and the total weight was recorded. Duplicate weighed portions (ca 0.05g) were taken for radioassay after oxidation. The extracts were combined and concentrated under a constant stream of nitrogen gas to near dryness and reconstituted in acetonitrile/0.1% aqueous formic acid, 1:1 (v/v) by vortex mixing, sonication and centrifugation at 10000 × g for 5 min at 4°C. The concentrates were weighed, and duplicate weighed aliquots (0.025 – 0.05 mL) taken for measurement of radioactivity to calculate recovery following concentration. An aliquot of each concentrated extract was injected into the HPLC.

For Liquid scintillation analyses, carcasses from rats (Group 2) were solubilized by digestion at ca 55ºC overnight in a solution prepared from sodium hydroxide (80 g) and Triton X-405 (100 mL) in purified water (600 mL) and methanol (300 mL). After measurement of total sample weight (as appropriate), replicate portions of diluted dose solutions (ca 0.1 mL), plasma (ca 0.05 – 0.25 mL), urine (ca 0.05 – 0.25 mL), expired air trap solution (ca 1 mL) and cage wash (ca 1 mL) were mixed directly with Ultima Gold scintillation cocktail (5 mL; 15 mL for air traps) for measurement of radioactivity concentrations. Similarly, replicate weighed portions of digested carcass solutions (ca 1 mL plus 1 mL purified water) were mixed with Ultima Gold (10 mL) scintillation cocktail for measurement of radioactivity concentrations. Feces were weighed, then homogenized to a smooth paste after the addition of an appropriate volume (approximately 1.5 volumes) of purified water and the weight of each homogenate was recorded. Replicate weighed portions of feces (ca 0.2 g) and blood cells (ca. 0.25 g) were placed on Combustocones, which were then capped with cellulose pads and burned in oxygen using

an Automatic Sample Oxidiser. The products of combustion were absorbed in Carbosorb E and mixed with Permafluor E+ for measurement of radioactivity concentrations.

Supplementary Figures

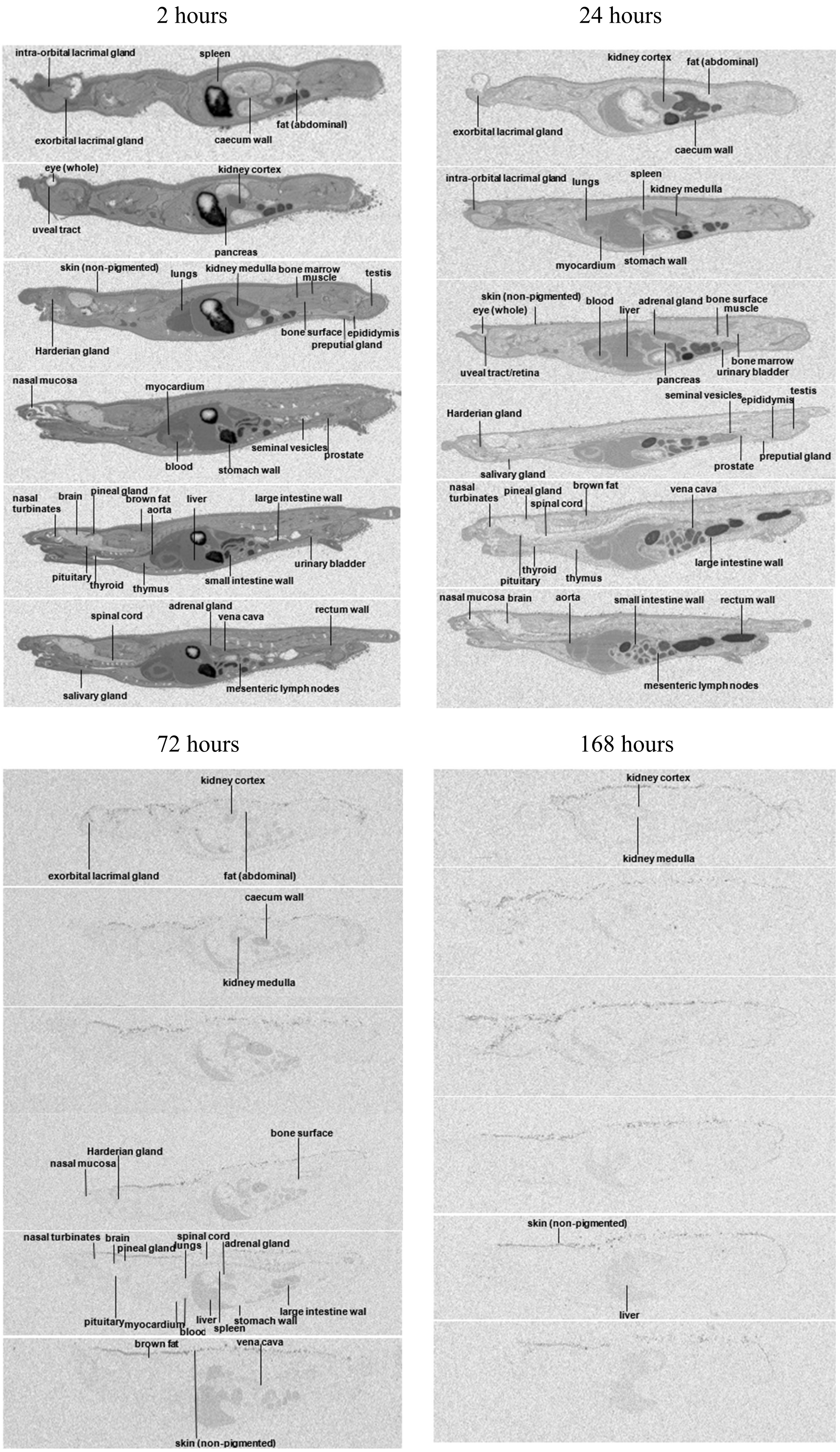

Supplementary Figure S1: Distribution of radioactivity at 2, 24, 72 and 168 hours following a single administration of ^{14}C-zapnometinib (30 mg/kg) to male albino rats. Whole body autoradiogram for one representative animal is shown.

0.5 hours

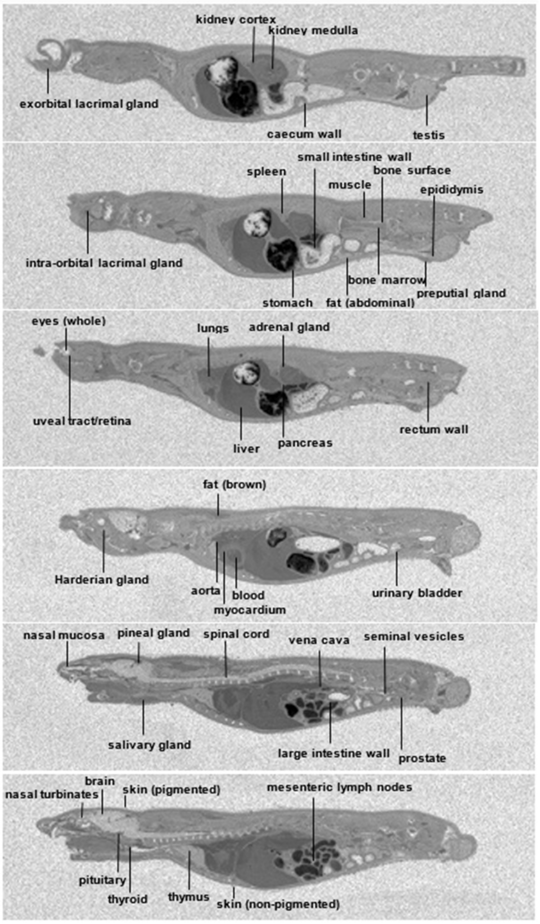

4 hours

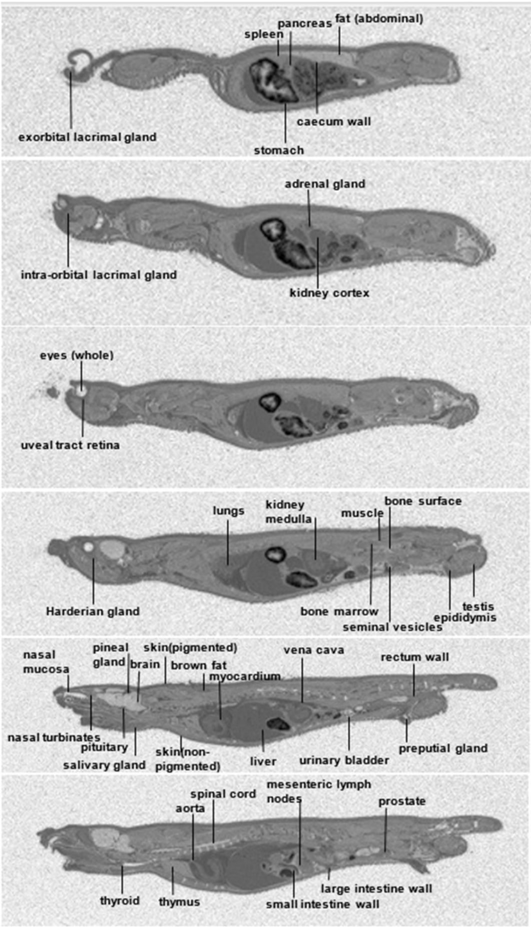

8 hours

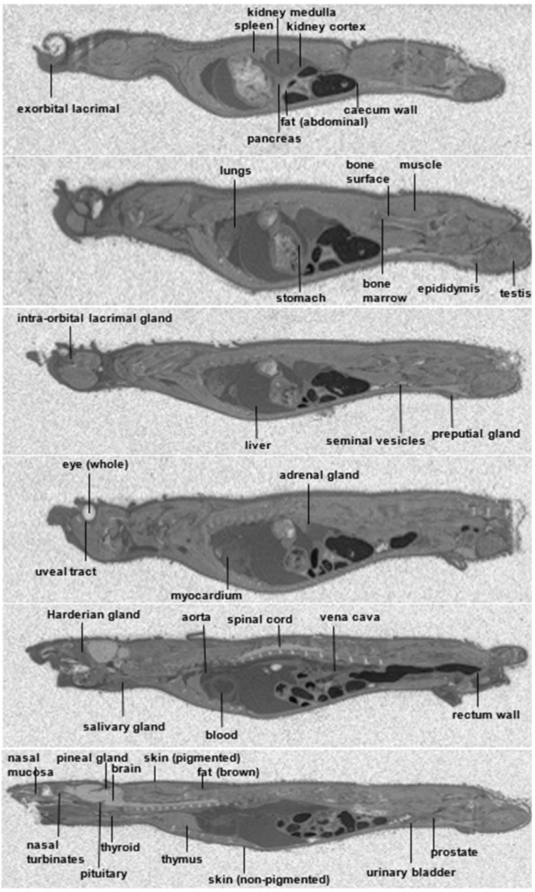

168 hours

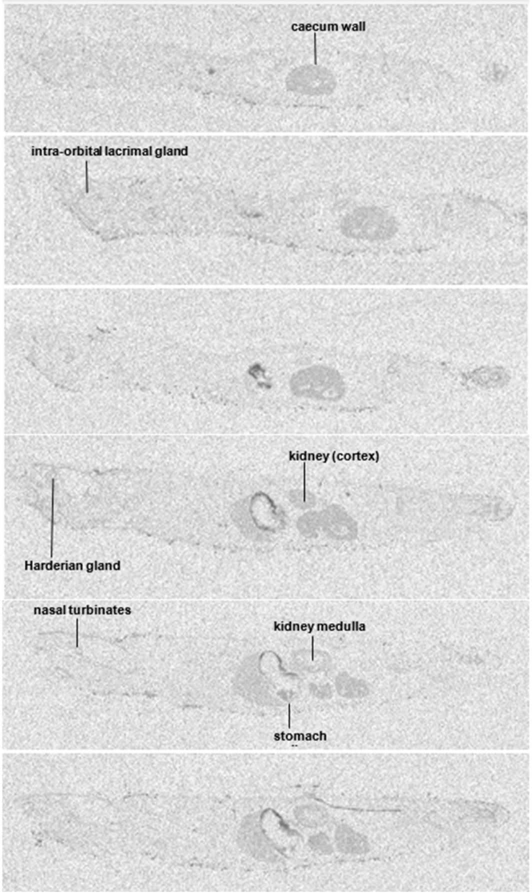

Supplementary Figure S2: Distribution of radioactivity at 0.5, 4, 8 and 168 hours following a single administration of ^{14}C-zapnometinib (30 mg/kg) to male partially pigmented rats. Whole body autoradiogram for one representative animal is shown.

0.5 hours

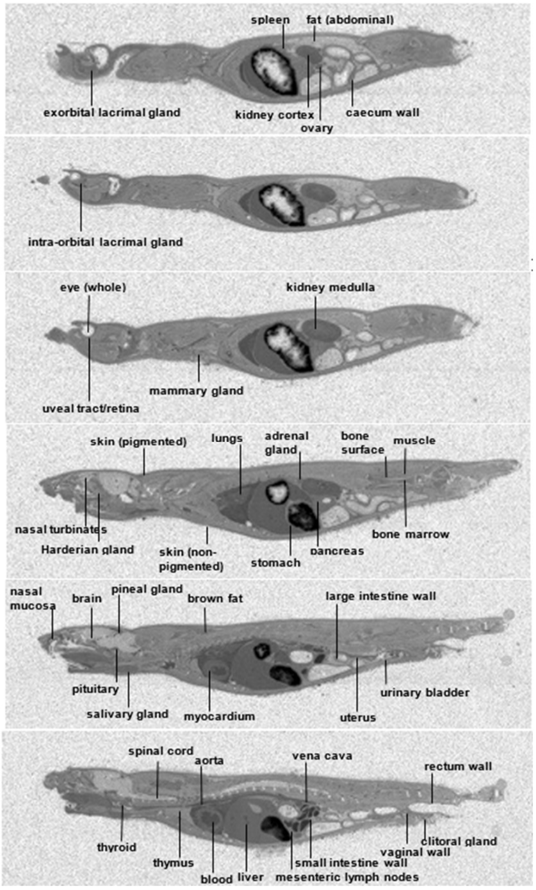

4 hours

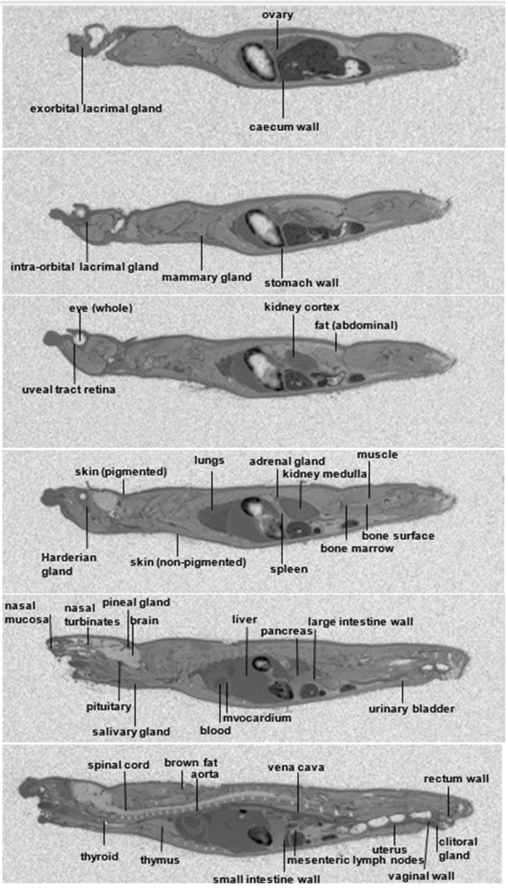

8 hours

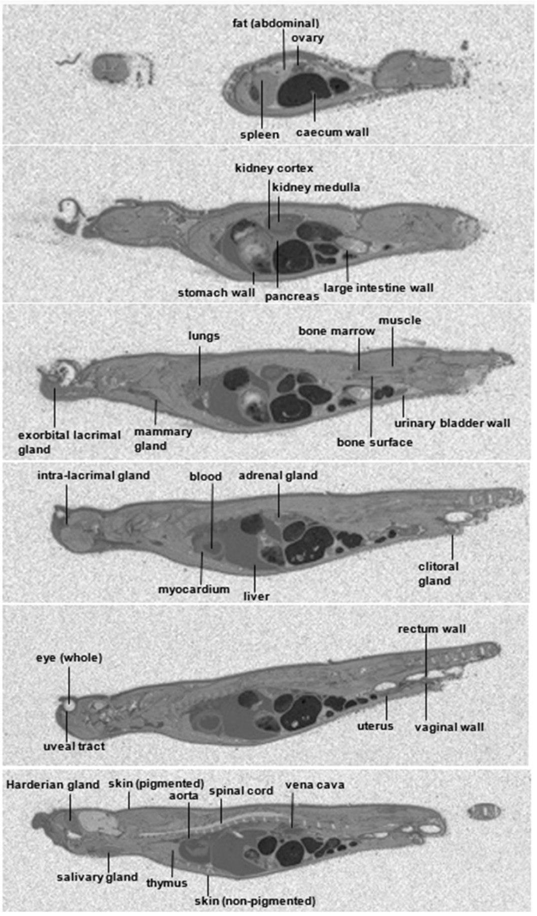

168 hours

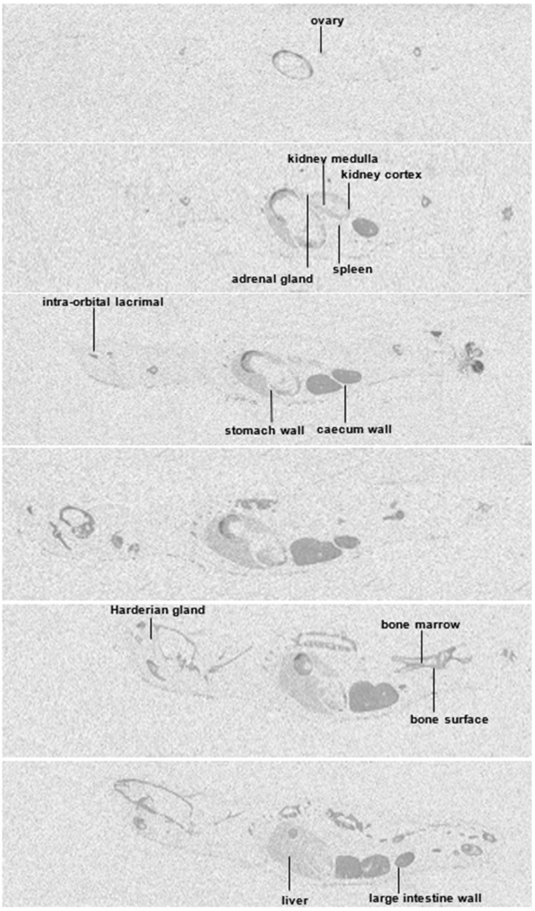

Supplementary Figure S3: Distribution of radioactivity at 0.5, 4, 8 and 168 hours following a single administration of ^{14}C-zapnometinib (30 mg/kg) to female partially pigmented rats. Whole body autoradiogram for one representative animal is shown.

A

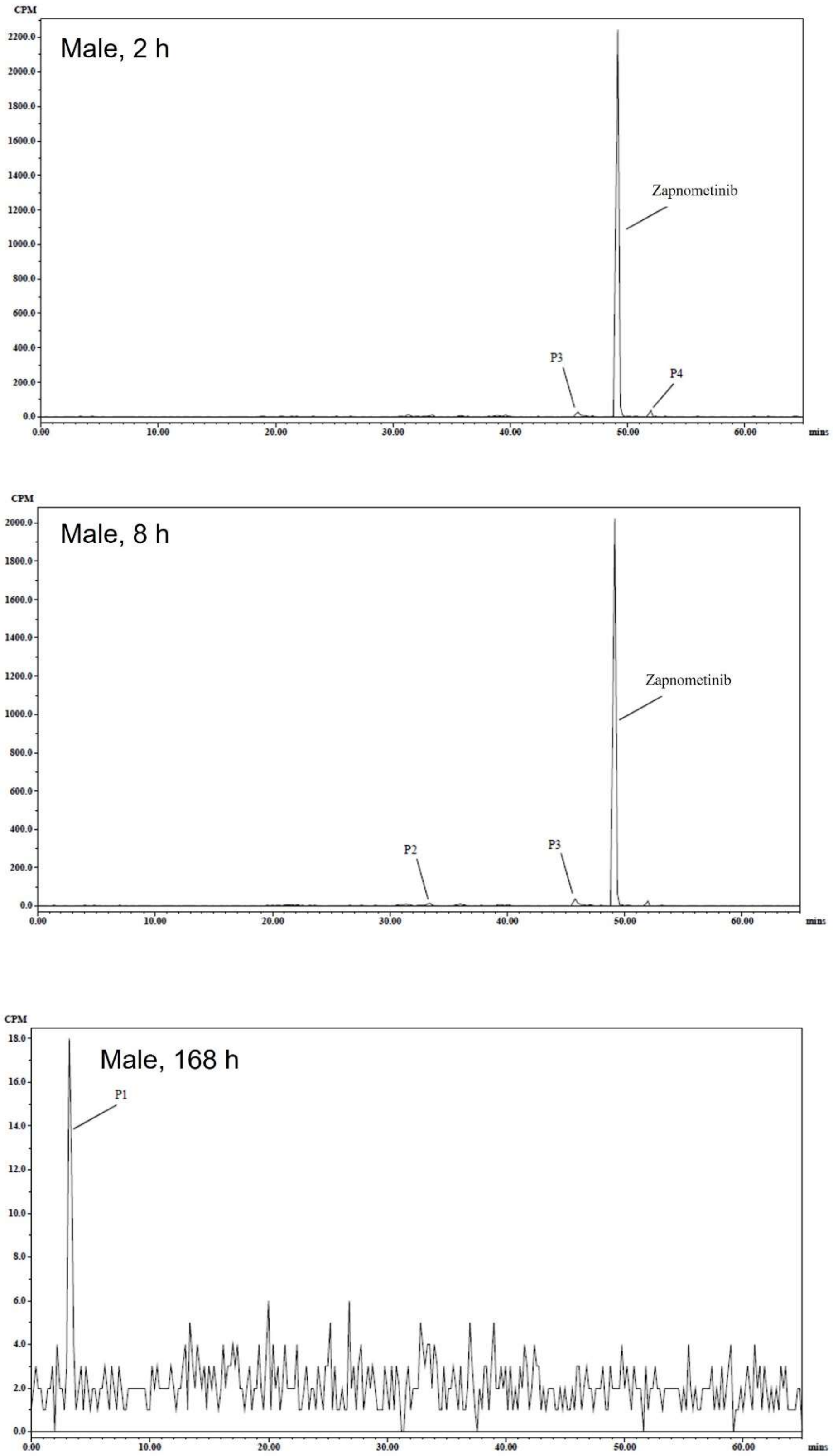

B

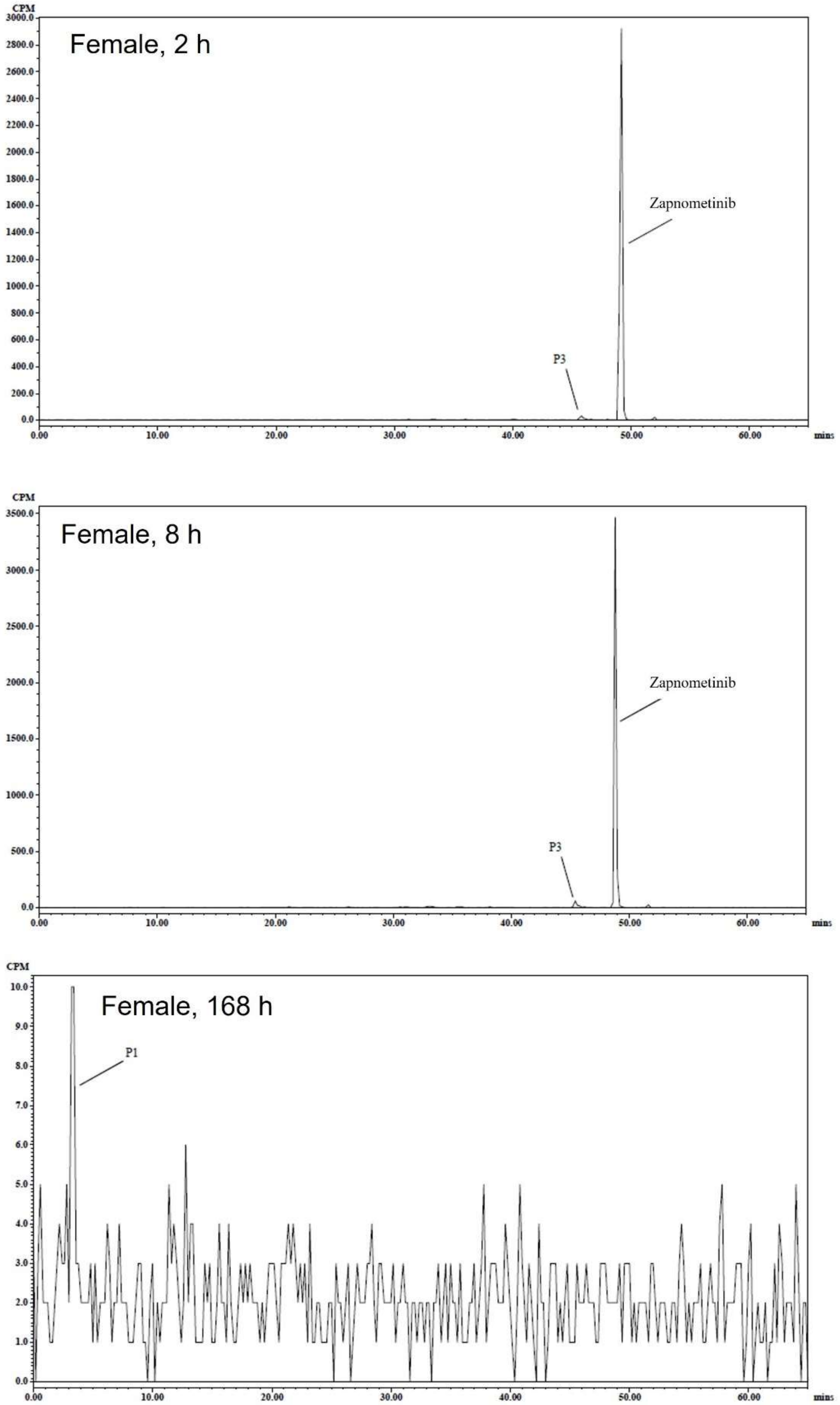

Supplementary Figure S4: Representative HPLC radiochromatograms of plasma extracts after 2, 8 and 168 hours following a single oral administration of [^{14}C]-zapnometinib (30 mg/kg) to male (A) and female (B) rats (Group 2).

A

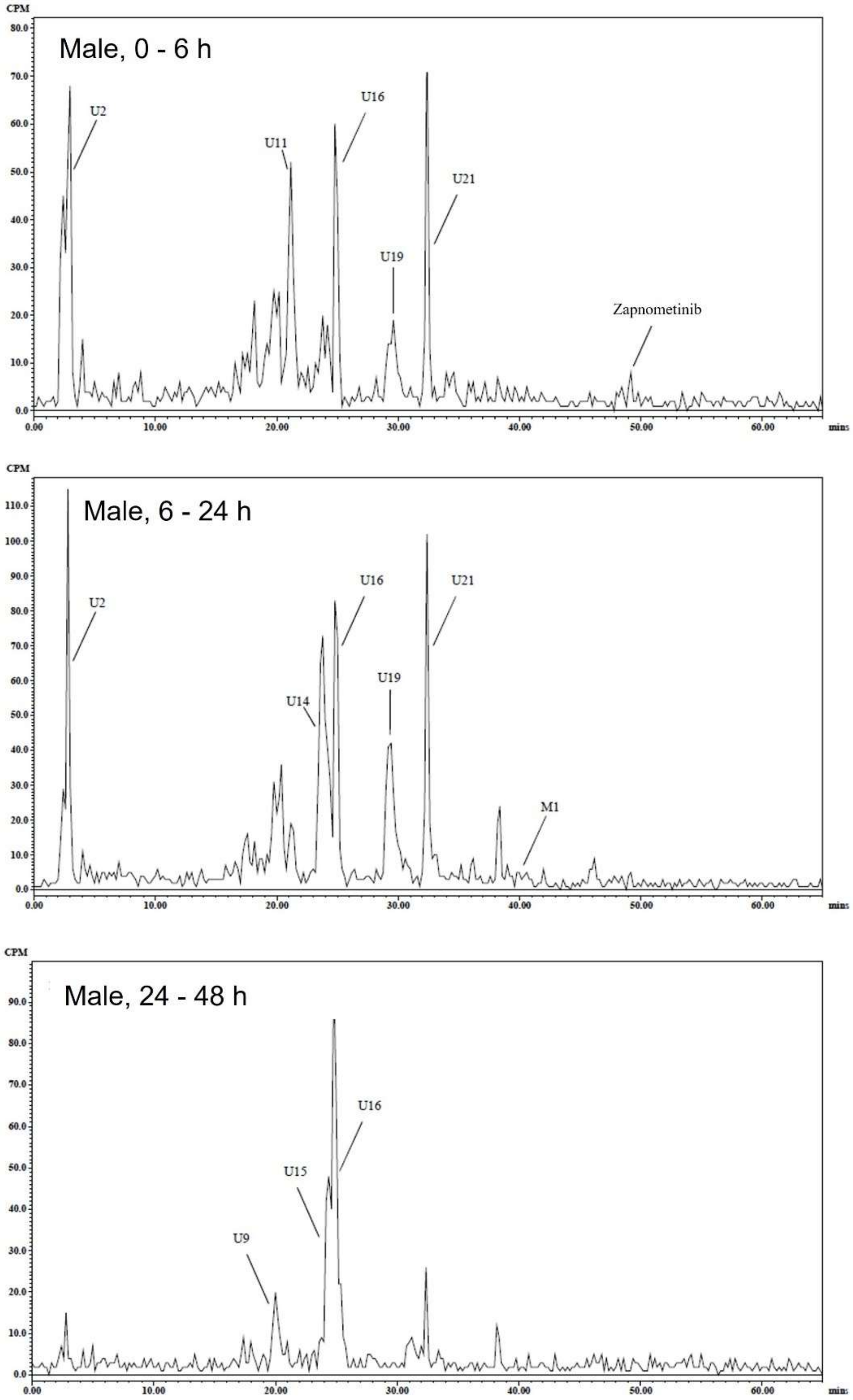
CPM
Male, 0 - 6 h
U2
U11
U16
U21
U19
Zapnometinib
mins
CPM
Male, 6 - 24 h
U2
U16
U21
U14
U19
M1
mins
CPM
Male, 24 - 48 h
U16
U15
U9
mins

B

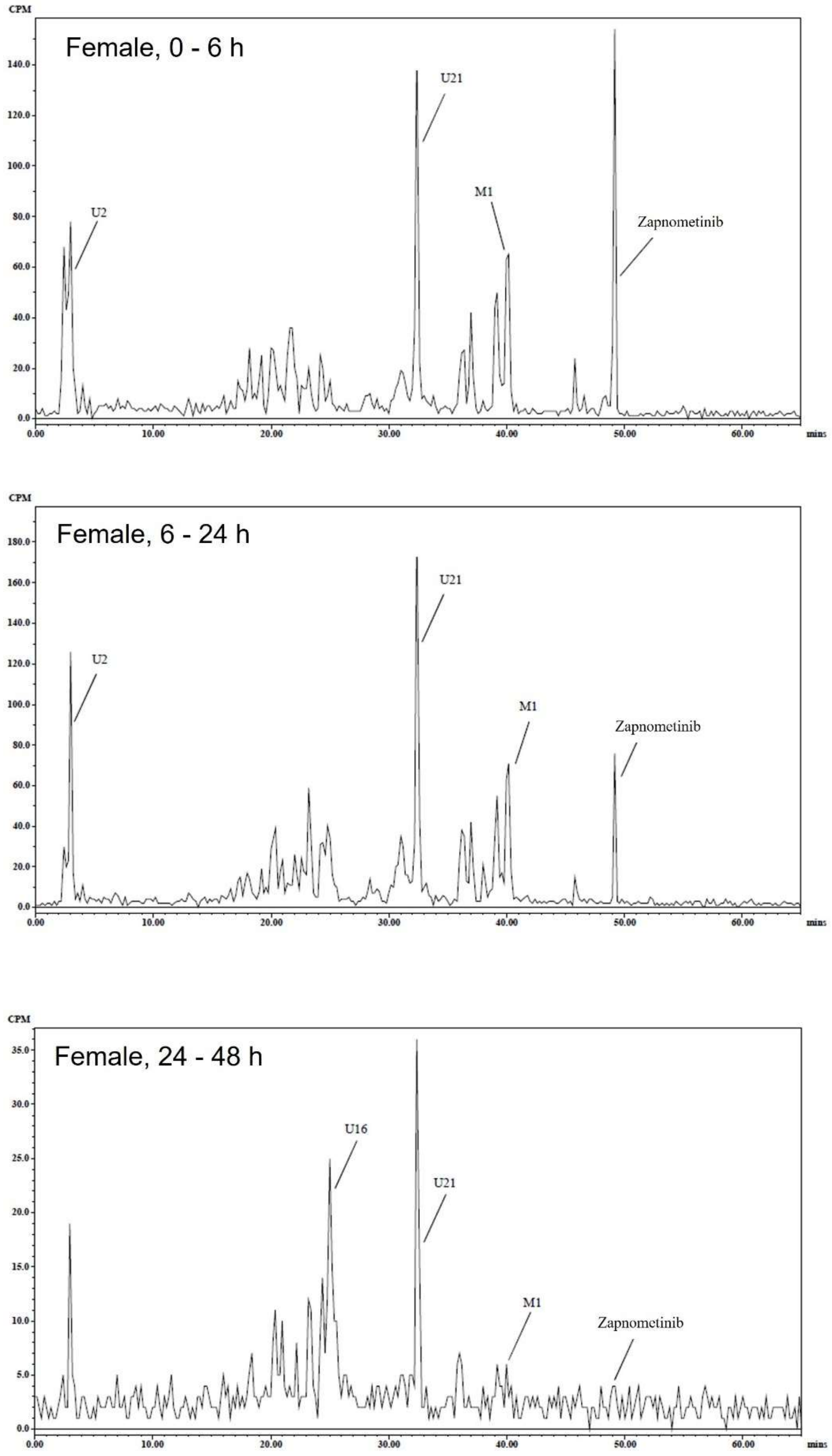

Supplementary Figure S5: Representative HPLC radiochromatograms of pooled urine from 0-6, 6 - 24 and 24 - 48 hours following a single oral administration of [^{14}C]-zapnometinib (30 mg/kg) to male (A) and female (B) rats (Group 2).

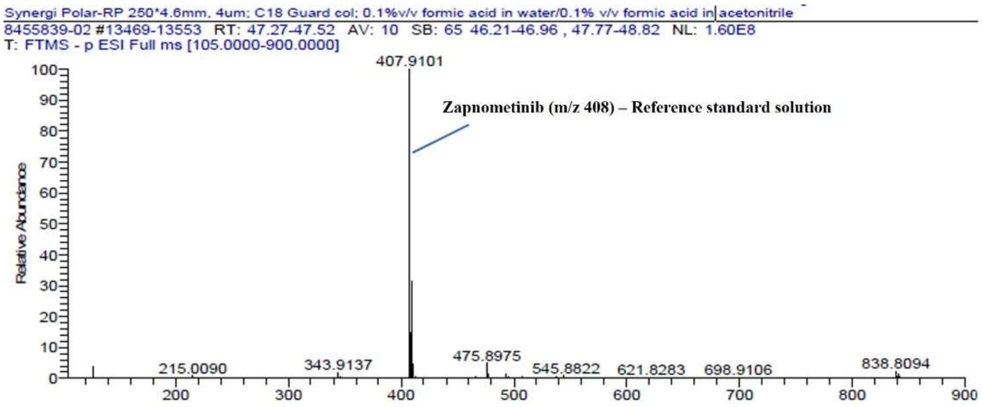
Synergi Polar-RP 250*4.6mm, 4um; C18 Guard col; 0.1%v/v formic acid in water/0.1% v/v formic acid in acetonitrile
8455839-02 #13469-13553 RT: 47.27-47.52 AV: 10 SB: 65 46.21-46.96 , 47.77-48.82 NL: 1.60E8
T: FTMS - p ESI Full ms [105.0000-900.0000]
407.9101
Zapnometinib (m/z 408) – Reference standard solution
215.0090
343.9137
475.8975
545.8822
621.8283
698.9106
838.8094
Relative Abundance

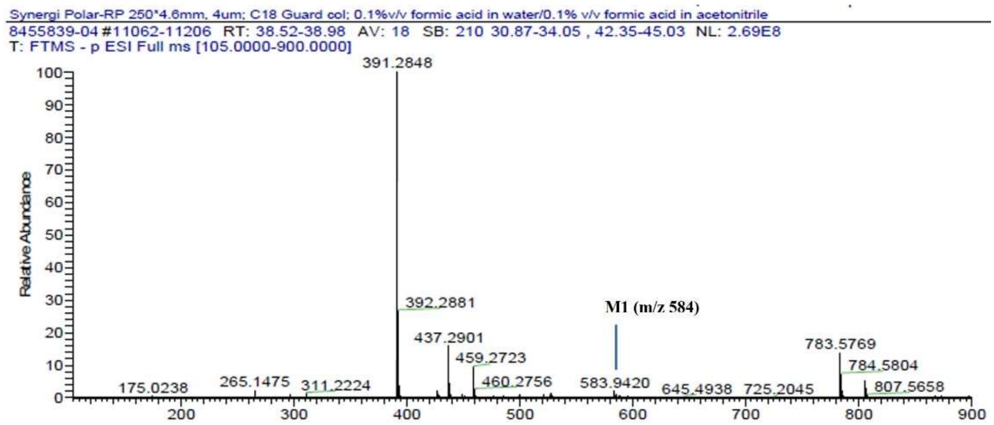
Synergi Polar-RP 250*4.6mm, 4um; C18 Guard col; 0.1%v/v formic acid in water/0.1% v/v formic acid in acetonitrile
8455839-04 #11062-11206 RT: 38.52-38.98 AV: 18 SB: 210 30.87-34.05 , 42.35-45.03 NL: 2.69E8
T: FTMS - p ESI Full ms [105.0000-900.0000]
391.2848
392.2881
M1 (m/z 584)
437.2901
459.2723
460.2756
583.9420
175.0238
265.1475
311.2224
645.4938
725.2045
783.5769
784.5804
807.5658
Relative Abundance

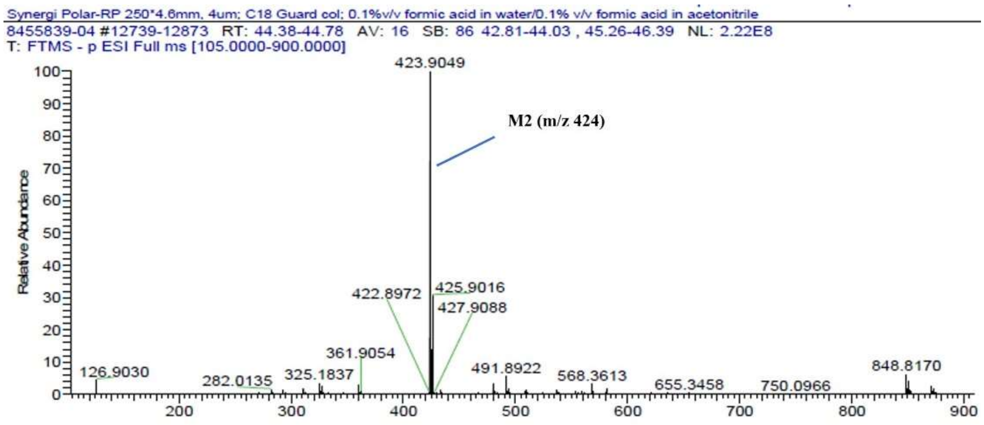
Synergi Polar-RP 250*4.6mm, 4um; C18 Guard col; 0.1%v/v formic acid in water/0.1% v/v formic acid in acetonitrile
8455839-04 #12739-12873 RT: 44.38-44.78 AV: 16 SB: 86 42.81-44.03 , 45.26-46.39 NL: 2.22E8
T: FTMS - p ESI Full ms [105.0000-900.0000]
423.9049
M2 (m/z 424)
422.8972
425.9016
427.9088
361.9054
126.9030
282.0135
325.1837
491.8922
568.3613
655.3458
750.0966
848.8170
Relative Abundance

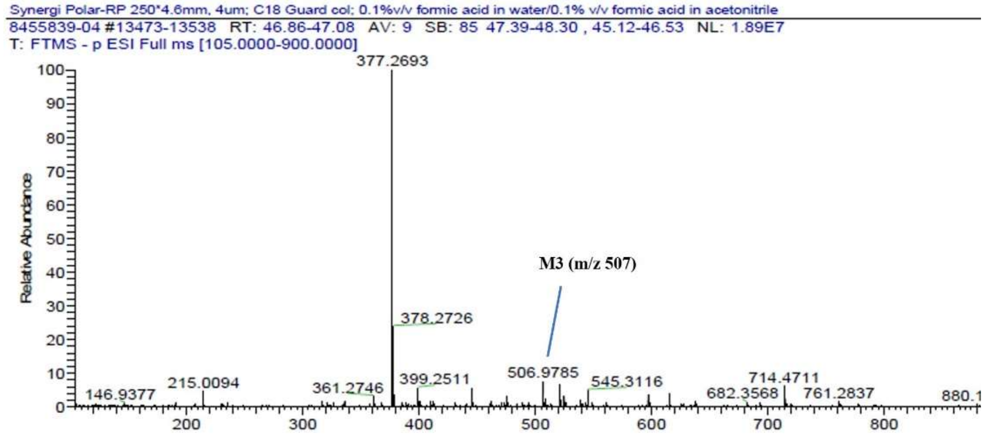
Synergi Polar-RP 250*4.6mm, 4um; C18 Guard col; 0.1%v/v formic acid in water/0.1% v/v formic acid in acetonitrile
8455839-04 #13473-13538 RT: 46.86-47.08 AV: 9 SB: 85 47.39-48.30 , 45.12-46.53 NL: 1.89E7
T: FTMS - p ESI Full ms [105.0000-900.0000]
377.2693
M3 (m/z 507)
378.2726
506.9785
146.9377
215.0094
361.2746
399.2511
545.3116
682.3568
714.4711
761.2837
880.1
Relative Abundance

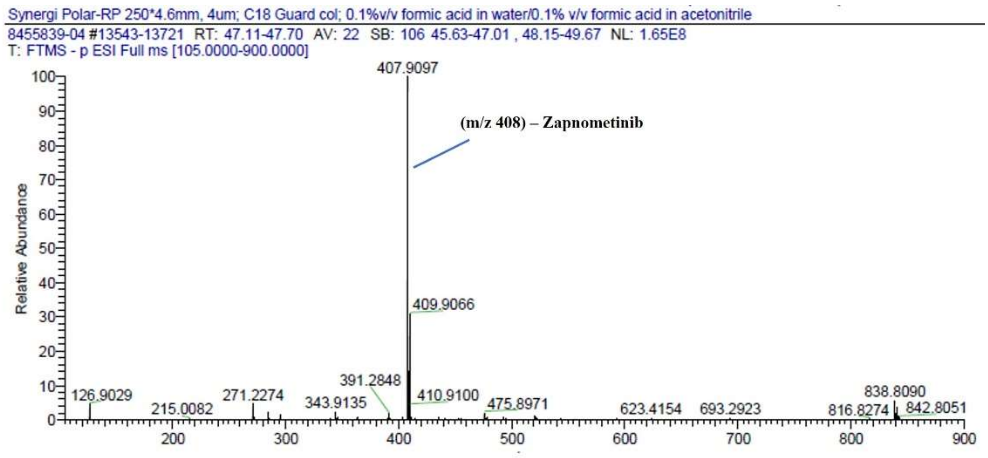
Synergi Polar-RP 250*4.6mm, 4um; C18 Guard col; 0.1%v/v formic acid in water/0.1% v/v formic acid in acetonitrile
8455839-04 #13543-13721 RT: 47.11-47.70 AV: 22 SB: 106 45.63-47.01 , 48.15-49.67 NL: 1.65E8
T: FTMS - p ESI Full ms [105.0000-900.0000]
407.9097
(m/z 408) – Zapnometinib
409.9066
391.2848
410.9100
126.9029
215.0082
271.2274
343.9135
475.8971
623.4154
693.2923
838.8090
816.8274
842.8051
Relative Abundance

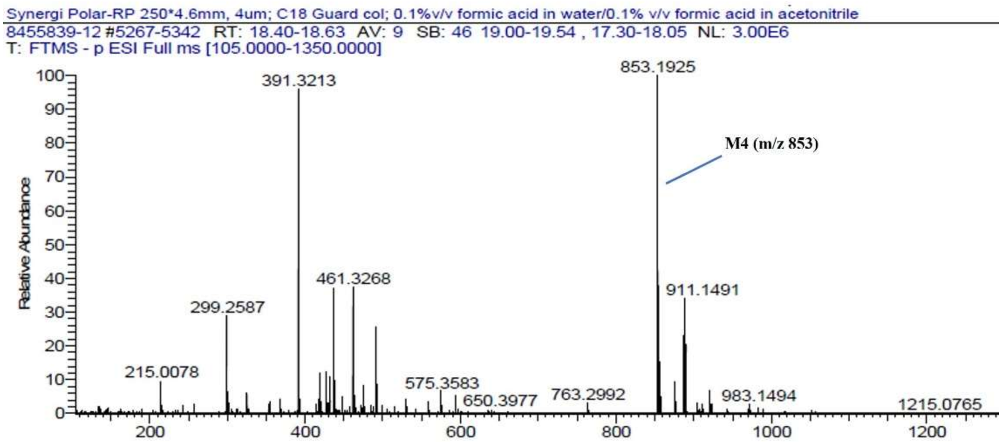
Synergi Polar-RP 250*4.6mm, 4um; C18 Guard col; 0.1%v/v formic acid in water/0.1% v/v formic acid in acetonitrile
8455839-12 #5267-5342 RT: 18.40-18.63 AV: 9 SB: 46 19.00-19.54 , 17.30-18.05 NL: 3.00E6
T: FTMS - p ESI Full ms [105.0000-1350.0000]
391.3213
853.1925
M4 (m/z 853)
461.3268
911.1491
299.2587
215.0078
575.3583
650.3977
763.2992
983.1494
1215.0765
Relative Abundance

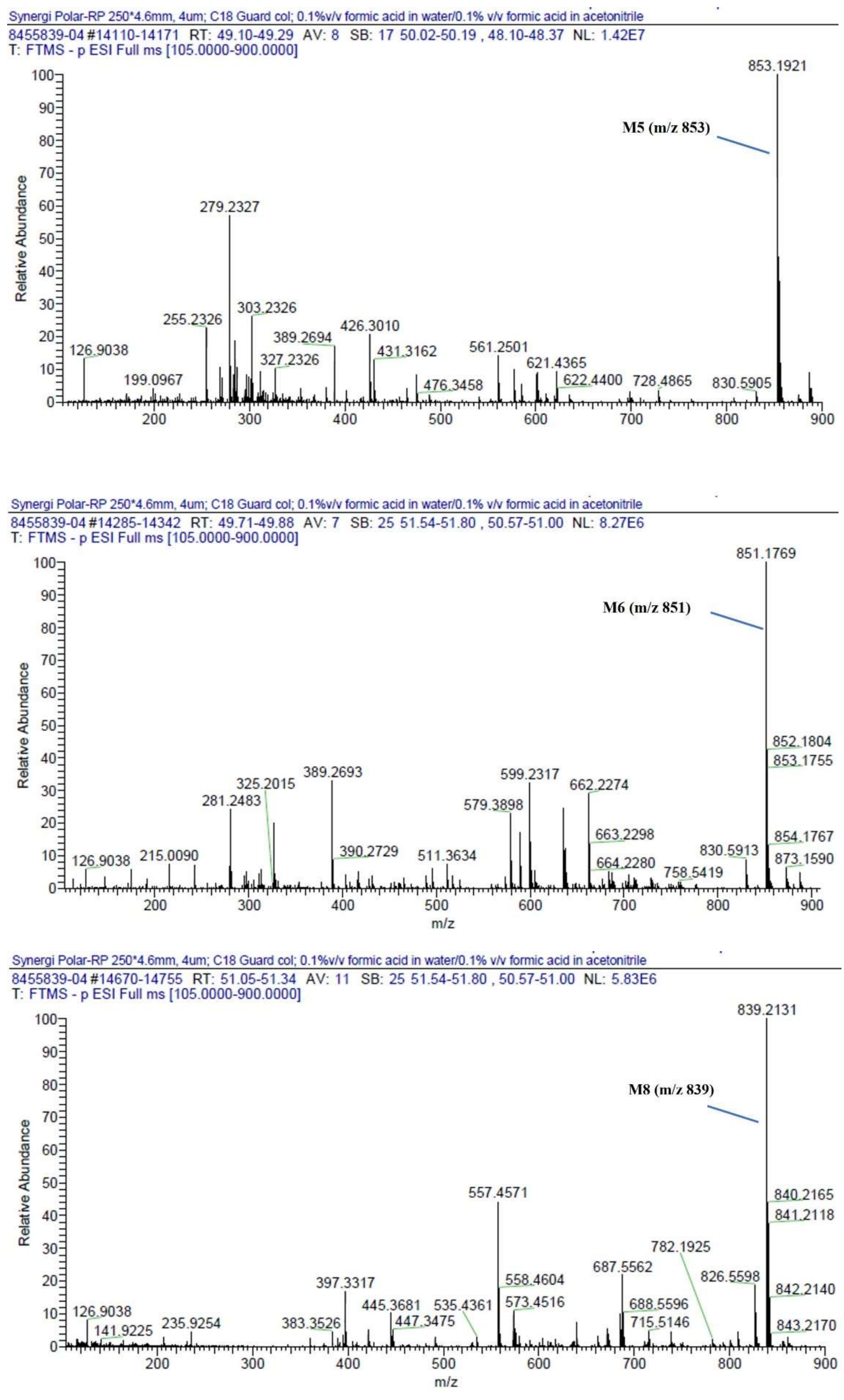

Supplementary Figure S6: Representative mass spectra from the LC-MS analyses in metabolite identification

Supplementary Tables

Supplementary Table S1: Pharmacokinetic parameters derived from total radioactivity in plasma following a single oral administration of [^{14}C]-zapnometinib (30 mg/kg) to male and female rats (Group 1)

Males

**Parameter**	**Concentration of radioactivity (µg equivalents/mL)**					
	**1M**	**2M**	**3M**	**Mean**	**SD**	**CV (%)**
C_{max} (µg equiv./mL)	89.2	81.2	79.4	83.3	5.22	6.26
T_{max} (h)	8.00	2.00	2.00	4.00	3.46	86.6
AUC_{0-t} (h*µg equiv./mL)	1530	1200	1020	1250	256	20.5
AUC_{0-inf} (h*µg equiv./mL)	1530	1200	1030	1260	256	20.4
$t_{1/2}$ (h)	35.1	28.3	39.8	34.4	5.79	16.8

Females

**Parameter**	**Concentration of radioactivity (µg equivalents/mL)**					
	**4F**	**5F**	**6F**	**Mean**	**SD**	**CV (%)**
C_{max} (µg equiv./mL)	130	107	130	122	13.3	10.9
T_{max} (h)	2.00	2.00	4.00	2.67	1.15	43.3
AUC_{0-t} (h*µg equiv./mL)	1830	1540	1940	1770	205	11.6
AUC_{0-inf} (h*µg equiv./mL)	1830	1540	1940	1770	205	11.6
$t_{1/2}$ (h)	37.2	36.0	23.4	32.2	7.66	23.8

SD Standard deviation, CV Coefficient of variance

Supplementary Table S2: Amounts (% Dose) of the principal components separated by HPLC in pooled urine following a single oral administration of [^{14}C]-zapnometinib (30 mg/kg) to male rats

Radioactive component	Retention time (minutes)	% Dose			
		0 – 6 hours	6 – 24 hours	24 – 48 hours	Total (0 – 48 hours)
U1	2.5	0.01	0.05	<0.01	0.06
U2	3.0	0.03	0.20	0.01	0.24
U3	4.0	<0.01	0.03	ND	0.03
U4	7.0	<0.01	0.04	ND	0.04
U5	16.5	<0.01	0.02	ND	0.02
U6	17.5	0.01	0.04	0.01	0.06
U7	18.0	0.01	0.05	0.01	0.07
U8	19.0	0.01	ND	ND	0.01
U9	20.0	0.01	0.07	0.04	0.12
U10	20.5	0.01	0.10	ND	0.11
U11	21.0	0.03	0.07	0.01	0.11
U12	22.0	<0.01	ND	<0.01	<0.01
U13	22.5	<0.01	ND	ND	<0.01
U14	23.5	0.01	0.34	<0.01	0.35
U15	24.5	0.01	ND	0.09	0.10
U16	25.0	0.02	0.21	0.21	0.44
U17	26.5	ND	0.02	ND	0.02
U18	28.5	<0.01	ND	ND	<0.01
U19	29.5	0.01	0.20	ND	0.21
U20	31.0	ND	0.03	0.02	0.05
U21	32.5	0.02	0.20	0.03	0.25
U22	34.0	0.01	ND	ND	0.01
U23	36.0	<0.01	0.02	ND	0.02
U24	37.0	ND	ND	ND	<0.01
U25	38.0	ND	0.05	0.01	0.06
U26	39.0	ND	ND	ND	ND
M1	40.0	ND	0.02	ND	0.02
U28	46.0	ND	0.02	ND	0.02
ATR-002	49.2	<0.01	ND	ND	<0.01
Remainder		0.02	0.18	0.03	0.23
Total		0.22	1.96	0.47	2.65

ND, Not detected
Remainder Accounts for areas on radiochromatogram which sum above zero but contain no discrete area of radioactivity

Supplementary Table S3: Amounts (% Dose) of the principal components separated by HPLC in pooled urine following a single oral administration of [^{14}C]-zapnometinib (30 mg/kg) to female rats

Radioactive component	Retention time (minutes)	% Dose			
		0 – 6 hours	6 – 24 hours	24 – 48 hours	Total (0 – 48 hours)
U1	2.5	0.02	0.04	ND	0.06
U2	3.0	0.06	0.23	0.05	0.34
U3	4.0	ND	ND	ND	ND
U4	7.0	ND	ND	ND	ND
U5	16.5	ND	ND	0.01	0.01
U6	17.5	0.01	0.04	ND	0.05
U7	18.0	0.01	0.07	0.02	0.10
U8	19.0	0.02	0.04	ND	0.06
U9	20.0	0.03	ND	ND	0.03
U10	20.5	ND	0.14	0.04	0.18
U11	21.0	ND	0.06	0.03	0.09
U12	22.0	0.04	0.09	0.02	0.15
U13	22.5	0.01	0.06	ND	0.07
U14	23.5	0.01	0.14	0.05	0.20
U15	24.5	0.01	0.09	0.04	0.14
U16	25.0	0.01	0.16	0.15	0.32
U17	26.5	ND	ND	0.02	0.02
U18	28.5	0.01	0.04	ND	0.05
U19	29.5	ND	ND	ND	ND
U20	31.0	0.03	0.19	0.02	0.24
U21	32.5	0.08	0.40	0.12	0.60
U22	34.0	ND	ND	ND	ND
U23	36.0	0.02	0.12	0.03	0.17
U24	37.0	0.02	0.10	ND	0.12
U25	38.0	ND	0.04	ND	0.04
U26	39.0	0.04	0.15	0.02	0.21
M1	40.0	0.05	0.23	0.01	0.29
U28	46.0	0.01	0.03	ND	0.04
ATR-002	49.2	0.06	0.10	0.01	0.17
Remainder		0.05	0.27	0.06	0.38
Total		0.60	2.83	0.70	4.13

ND, Not detected
Remainder Accounts for areas on radiochromatogram which sum above zero but contain no discrete area of radioactivity

Supplementary Table S4: Amounts (% Dose) of the principal components separated by HPLC in pooled feces extracts following a single oral administration of [^{14}C]-zapnometinib (30 mg/kg) to male rats (Group 2)

Radioactive component	Retention time (minutes)	% Dose		
		0 – 24 hours	24 – 48 hours	Total (0 – 48 hours)
F1	36.0	ND	0.13	0.13
F2	28.0	ND	0.21	0.21
F3	39.0	1.03	0.21	1.24
M1	40.0	1.59	ND	1.59
M2	46.0	9.76	2.22	11.98
M3	49.0	4.21	ND	4.21
Zapnometinib	49.2	19.28	2.15	21.48
M4	50.0	3.73	1.49	5.22
M5	51.0	18.33	1.65	19.98
M6	52.0	11.66	0.66	12.32
M7	52.5	1.59	0.46	2.05
M8	53.0	4.36	0.34	4.70
	Remainder	2.54	0.48	3.02
	Total	79.34	9.98	89.32

ND — Not detected

Remainder — Accounts for areas on radiochromatogram which sum above zero but contain no discrete area of radioactivity

Supplementary Table S5: Amounts (% Dose) of the principal components separated by HPLC in pooled feces extracts following a single oral administration of [^{14}C]-zapnometinib (30 mg/kg) to female rats (Group 2)

Radioactive component	Retention time (minutes)	% Dose		
		**0 – 24 hours**	**24 – 48 hours**	**Total (0 – 48 hours)**
F1	36.0	0.69	0.44	1.13
F2	28.0	0.75	0.33	1.08
F3	39.0	ND	ND	ND
M1	40.0	1.10	0.27	1.37
M2	46.0	9.32	6.32	15.64
M3	49.0	16.10	2.48	18.58
Zapnometinib	49.2	19.87	6.30	26.17
M4	50.0	3.22	2.08	5.30
M5	51.0	3.84	1.35	5.19
M6	52.0	3.70	0.38	4.08
M7	52.5	4.11	1.15	5.26
M8	53.0	2.06	0.69	2.75
	Remainder	3.75	0.32	4.07
	Total	68.51	22.11	90.62

ND Not detected

Remainder Accounts for areas on radiochromatogram which sum above zero but contain no discrete area of radioactivity

Supplementary Table S6: Concentrations and proportions of the principal components separated by HPLC in extracts of plasma in male rats

Radioactive component	Retention time (minutes)	µg equivalents zapnometinib/g (% sample)		
		2 hours	8 hours	168 hours
P1	3.0	ND	ND	0.043 (68.8)
P2	33.0	ND	0.779 (1.0)	0.018 (29.5)
P3	46.0	1.26 (1.8)	1.87 (2.4)	ND
zapnometinib	49.2	64.4 (92.0)	72.1 (92.5)	ND
P4	52.0	0.980 (1.4)	0.779 (1.0)	ND
	Remainder	3.36 (4.8)	2.42 (3.1)	0.001 (1.7)
	Total	70.0 (100)	77.9 (100)	0.062 (100)

ND — Not detected

Remainder — Accounts for areas on radiochromatogram which sum above zero but contain no discrete area of radioactivity

Supplementary Table S7: Concentrations and proportions of the principal components separated by HPLC in extracts of plasma in female rats

Radioactive component	Retention time (minutes)	µg equivalents zapnometinib/g (% sample)		
		2 hours	8 hours	168 hours
P1	3.0	ND	ND	0.632 (51.0)
P2	33.0	ND	0.91 (1.0)	ND
P3	46.0	1.20 (1.5)	2.18 (2.4)	ND
zapnometinib	49.2	78.4 (97.9)	80.7 (88.7)	ND
P4	52.0	ND	0.637 (0.7)	ND
	Remainder	0.481 (0.6)	6.55 (7.2)	0.608 (49.0)
	Total	80.1 (100)	91.0 (100)	1.24 (100)

ND — Not detected

Remainder — Accounts for areas on radiochromatogram which sum above zero but contain no discrete area of radioactivity

Supplementary Table S8: Cumulative excretion of radioactivity following a single oral administration of [^{14}C]-zapnometinib (30 mg/kg) to male rats (Group 2)

Sample and collection period (h)	% administered dose				
	7M	8M	9M	Mean	SD
Urine					
0 – 6	0.23	0.16	0.30	0.23	0.07
0 – 24	2.69	1.99	1.92	2.20	0.43
0 – 48	3.13	2.48	2.43	2.68	0.39
0 – 72	3.20	2.55	2.56	2.77	0.37
0 – 96	3.22	2.58	2.61	2.80	0.36
0 – 120	3.23	2.59	2.63	2.82	0.36
0 – 144	3.24	2.60	2.64	2.83	0.36
0 – 168	3.24	2.60	2.65	2.83	0.36
Faeces					
0 – 24	81.33	77.94	78.75	79.34	1.77
0 – 48	90.61	88.87	88.48	89.32	1.13
0 – 72	91.30	89.52	89.53	90.12	1.02
0 – 96	91.37	89.65	89.82	90.28	0.95
0 – 120	91.41	89.69	89.92	90.34	0.93
0 – 144	91.44	89.72	89.96	90.37	0.93
0 – 168	91.46	89.89	89.99	90.45	0.88
Cage wash					
0 – 24	0.14	0.16	0.11	0.14	0.03
0 – 48	0.22	0.19	0.14	0.18	0.04
0 – 72	0.23	0.20	0.15	0.19	0.04
0 – 96	0.23	0.24	0.16	0.21	0.04
0 – 120	0.23	0.24	0.16	0.21	0.04
0 – 144	0.23	0.24	0.16	0.21	0.04
0 – 168	0.23	0.26	0.17	0.22	0.05
Expired air					
0 – 24	0.02	0.02	0.02	0.02	0.00
0 – 48	0.02	0.02	0.02	0.02	0.00
0 – 72	0.02	0.02	0.02	0.02	0.00

SD Standard deviation

Supplementary Table S9: Cumulative excretion of radioactivity following a single oral administration of [^{14}C]-zapnometinib (30 mg/kg) to female rats (Group 2)

Sample and collection period (h)	% administered dose				
	**10F**	**11F**	**12F**	**Mean**	**SD**
Urine					
0 – 6	0.44	0.35	0.28	0.36	0.08
0 – 24	3.09	3.03	3.50	3.21	0.26
0 – 48	3.79	3.70	4.26	3.92	0.30
0 – 72	3.96	3.81	4.37	4.05	0.29
0 – 96	4.00	3.84	4.41	4.08	0.29
0 – 120	4.02	3.86	4.43	4.10	0.29
0 – 144	4.03	3.87	4.44	4.11	0.29
0 – 168	4.04	3.88	4.45	4.12	0.29
Faeces					
0 – 24	68.79	74.70	62.03	68.51	6.34
0 – 48	90.11	96.59	85.16	90.62	5.73
0 – 72	96.05	97.23	85.96	93.08	6.19
0 – 96	96.19	97.39	86.06	93.21	6.22
0 – 120	96.25	97.44	86.10	93.26	6.23
0 – 144	96.29	97.47	86.13	93.30	6.23
0 – 168	96.32	97.49	86.15	93.32	6.24
Cage wash					
0 – 24	0.39	0.21	0.35	0.32	0.09
0 – 48	0.48	0.37	0.42	0.42	0.06
0 – 72	0.50	0.39	0.44	0.44	0.06
0 – 96	0.51	0.40	0.45	0.45	0.06
0 – 120	0.52	0.40	0.45	0.46	0.06
0 – 144	0.53	0.40	0.46	0.46	0.07
0 – 168	0.85	0.42	0.53	0.60	0.22
Expired air					
0 – 24	0.02	0.02	0.02	0.02	0.00
0 – 48	0.02	0.02	0.02	0.02	0.00
0 – 72	0.02	0.02	0.02	0.02	0.00

SD Standard deviation
BLQ Below the limit of quantification (<2 × background radioactivity)

Supplementary Table S10: Concentrations of radioactivity in tissues determined by QWBA following a single oral administration of [^{14}C]- zapnometinib (30 mg/kg) to male albino rats (Group 4)

Tissue type	Tissue/organ	Concentrations of total radioactivity (µg equivalents Zapnometinib/g)			
		**27M 2 h**	**28M 24 h**	**29M 72 h**	**30M 168 h**
Non QWBA	Plasma [a]	**48.2**	4.24	0.185	0.076
	Blood cells [b]	**6.00**	0.314	0.050	0.042
Circulatory	Aorta	**67.2**	3.94	BLQ	BLQ
	Blood (cardiac)	**96.8**	8.11	0.555	BLQ
	Vena cava	**47.3**	1.39	0.386	BLQ
Nervous	Brain	**3.12**	0.297	0.370	BLQ
	Spinal cord	**4.20**	0.264	0.306	BLQ
Ocular	Eye (whole)	**0.568**	0.557	BLQ	BLQ
	Uveal tract/retina	**23.6**	2.86	BLQ	BLQ
Visceral	Kidney cortex	**67.3**	7.87	0.916	0.406
	Kidney medulla	**37.0**	4.31	0.456	0.257
	Liver	**66.4**	7.04	1.27	0.513
	Lungs	**51.0**	4.80	0.520	BLQ
	Myocardium	**46.2**	2.49	0.276	BLQ
	Spleen	**15.9**	1.26	0.338	BLQ
Glandular/ Secretory	Adrenal gland	**33.0**	3.80	0.490	BLQ
	Exorbital lacrimal gland	**16.4**	1.42	0.407	BLQ
	Harderian gland	**19.4**	3.39	0.582	BLQ
	Intra-orbital lacrimal gland	**70.5**	3.83	BLQ	BLQ
	Mesenteric lymph nodes	**12.1**	1.09	BLQ	BLQ
	Nasal mucosa	**7.58**	0.310	0.392	BLQ
	Nasal turbinates	**12.9**	1.22	0.454	BLQ
	Pancreas	**24.5**	1.77	BLQ	BLQ
	Pineal gland	**35.9**	2.67	0.593	BLQ
	Pituitary	**40.9**	1.87	0.448	BLQ
	Salivary gland	**43.8**	1.89	BLQ	BLQ
	Thymus	**15.3**	0.828	BLQ	BLQ
	Thyroid	**35.0**	2.04	BLQ	BLQ
Musculo-skeletal	Bone marrow	**14.9**	1.29	BLQ	BLQ
	Bone surface [c]	**26.0**	2.32	1.63	BLQ
	Fat (abdominal) [c]	**7.44**	0.721	0.426	BLQ
	Fat (brown)	**33.8**	1.64	0.451	BLQ
	Muscle	**15.6**	0.735	BLQ	BLQ
	Skin	**12.4**	4.97	2.26	1.75
Reproductive	Epididymis	**15.6**	2.42	BLQ	BLQ
	Preputial gland	**22.0**	1.41	BLQ	BLQ
	Prostate	**25.7**	2.78	BLQ	BLQ
	Seminal vesicles	**8.02**	0.771	BLQ	BLQ
	Testis	**13.1**	1.26	BLQ	BLQ
Excretory	Caecum wall	**22.1**	3.10	0.474	BLQ
	Large intestine wall	**29.9**	6.10	0.591	BLQ
	Rectum wall	**19.6**	8.09	BLQ	BLQ
	Small intestine wall	**32.4**	4.02	BLQ	BLQ
	Stomach wall	**25.1**	1.24	0.322	BLQ
	Urinary bladder wall	**25.1**	2.80	BLQ	BLQ

For all QWBA measurements:
Upper and lower limits of quantification = 510 and 0.118 µg equiv zapnometinib /g of tissue, respectively
QWBA Quantitative whole-body autoradiography
BLQ Tissue radioactivity concentration below the lower limit of quantification (LLOQ)
[a] Determined by direct liquid scintillation analysis (LLOQ = 0.007 – 0.013 µg equiv zapnometinib /g)
[b] Determined by oxidation and liquid scintillation analysis (LLOQ = 0.011 – 0.013µg equiv zapnometinib /g)
[c] Tissue corrected for quenching

Values in bold represent maximum tissue concentrations (T_{max})
